# Exact results of the limited penetrable horizontal visibility graph associated to random time series and its application

**DOI:** 10.1038/s41598-018-23388-1

**Published:** 2018-03-23

**Authors:** Minggang Wang, André L. M. Vilela, Ruijin Du, Longfeng Zhao, Gaogao Dong, Lixin Tian, H. Eugene Stanley

**Affiliations:** 1 School of Mathematical Science, Nanjing Normal University, Nanjing, 210042 Jiangsu China; 2 Department of Mathematics, Nanjing Normal University Taizhou College, Taizhou, 225300 Jiangsu China; 3 Center for Polymer Studies and Department of Physics, Boston University, Boston, MA 02215 USA; 4 Universidade de Pernambuco, 50720-001 Recife, PE Brazil; 5 Energy Development and Environmental Protection Strategy Research Center, Jiangsu University, Zhenjiang, 212013 Jiangsu China

## Abstract

The limited penetrable horizontal visibility algorithm is an analysis tool that maps time series into complex networks and is a further development of the horizontal visibility algorithm. This paper presents exact results on the topological properties of the limited penetrable horizontal visibility graph associated with independent and identically distributed (*i:i:d:*) random series. We show that the *i.i.d:* random series maps on a limited penetrable horizontal visibility graph with exponential degree distribution, independent of the probability distribution from which the series was generated. We deduce the exact expressions of mean degree and clustering coefficient, demonstrate the long distance visibility property of the graph and perform numerical simulations to test the accuracy of our theoretical results. We then use the algorithm in several deterministic chaotic series, such as the logistic map, H´enon map, Lorenz system, energy price chaotic system and the real crude oil price. Our results show that the limited penetrable horizontal visibility algorithm is efficient to discriminate chaos from uncorrelated randomness and is able to measure the global evolution characteristics of the real time series.

## Introduction

Several methodologies for understanding the complicated behavior of nonlinear time series have been recently developed, including chaos analysis^1,2^, fractal analysis^3,4^, and complexity measurement^5,6^. With the development of complex network theories^7–10^, a new multidisciplinary methodology for characterizing nonlinear time series using complex network science has emerged and rapidly expanded^11–26^. The main tool of these methods is to use an algorithm to transform a nonlinear time series into a corresponding complex network and then use the topological structure of complex networks to analyze the properties of the nonlinear time series.

Currently there are several ways of converting univariate time series into complex networks. The first one is Pseudo-periodic time series transitions^11^ that analyze pseudo-periodic time series. The second one is the visibility graph (VG) method, which was first proposed by Lacasa *et al*.^12,13^ and simplified by Luque *et al*.^14,15^. Bezsudnov *et al*.^16^ proposed a parameter visibility method and Gao *et al*.^17^ also proposed a limited penetrable visibility method (LPVG) and multiscale limited penetrable horizontal visibility graph (MLPHVG). The third one is the phase space reconstruction method^18,19^. It begins with a phase space reconstruction of time series analysis, maps fixed-length time series segments into nodes of a network, and then uses the correlation coefficients (or distances) between these nodes to determine whether they are connected or not. The fourth one is recurrence networks method^20,21^. This method uses the concept of recurrences in phase space and the recurrence matrix of a time series is interpreted as the adjacency matrix of an associated complex network. The links of this network connects different points in time if the considered states are closely neighboured in phase space. And the last one is the coarse graining method^22–25^ by which fluctuations of time series are transformed into signal sequences. A fixed-length signal sequence is treated as a network node that connects nodes of time series in chronological order, and a weighted and directed network is then constructed. In recent years, researchers have also used complex network theories to study multivariate time series^26–29^. These methods all effectively maintain most of the properties of different types of time series, and they have been successfully used in many different fields^30–37^.

Although there have been abundant empirical results obtained using time series complex network algorithms^11–29^, rigorous theoretical results are still lacking. Only a small amount of literature has presented exact results on the properties of the horizontal visibility graphs (HVG) associated with random series^12–15^. Thus far no rigorous theory other than the above algorithms have been developed. Our goal here is to focus on one type of general horizontal visibility algorithm, the limited penetrable horizontal visibility graph (LPHVG). The parameter *ρ* of the limited penetrable horizontal visibility graph is called limited penetrable distance, when we set the limited penetrable distance *ρ* = 0, the limited penetrable horizontal visibility graph (LPHVG) degenerates into the horizontal visibility graph (HVG)^14^, thus LPHVG is the extended form of HVG. We derive exact results on the properties of the limited penetrable horizontal visibility graphs associated with independent and identically distributed (*i.i.d*.) random series. We prove that an *i.i.d*. random series can be mapped on a limited penetrable horizontal visibility graph with exponential degree distribution, which is an extension of the result presented by Luque *et al*.^14^. We deduce the exact mean degree and the clustering coefficient, and we prove that the limited penetrable horizontal visibility graph associated with any *i.i.d*. random series has small-world characteristics. To verify our theoretical solution, we acquire simulation results by using several deterministic chaotic series and a real crude oil price series that confirms the accuracy and usability of our results.

## Results

Here we show several exact results of LPHVG associated with *i.i.d*. random time series and apply them to the deterministic chaotic series of a logistic map, a Hénon map, a Lorenz system and energy price chaotic system and a crude oil price series.

### Degree distribution

Let *X*(*t*) be a real valued bi-infinite time series of *i.i.d*. random variables with a probability density *f*(*x*) in which *x* ∈ [*a*, *b*], and consider its associated LPHVG with the limited penetrable distance *ρ* = 1. Then

1 $$P(k)\sim exp[-(k-\mathrm{4)}ln\mathrm{(5/4)}]\quad {\rm{with}}\quad k=4,5,\ldots ,\forall f(x),$$

where *P*(*k*) is the degree distribution and *k* is the degree of a node. To prove this conclusion we first calculate the probability that an arbitrary datum with value *x*_0_ has a limited penetrability at most a one-time visibility of *k* other data. We thus list all sets of possible configurations for data *x*_0_ with *k* = 4, 5 and 6 (Figs S1, S2 and S3 in Supplementary Information), and we calculate the probability *P*(*k* = 4), *P*(*k* = 5) and *P*(*k* = 6) (Eqs (S4), (S9) and (S10), respectively). We then deduce the rules of when a given configuration contributes to *P*(*k*) (rules i–iv in Supplementary Information) and obtain a general expression for *P*(*k*) [see Eq. (S12)]. The detailed proof of this result is shown in *Supplementary Information Theorem S1*. This is an exact result for a limited penetrable horizontal visibility graph with the limited penetrable distance *ρ* = 1. We conclude that for every probability distribution *f*(*x*), the degree distribution *P*(*k*) of the associated LPHVG has the same exponential form. In addition, from this result we can obtain the more general result (*Theorem S2* in *Supplementary Information*) in which *X*(*t*) is a real bi-infinite time series of *i.i.d*. random variables with a probability distribution *f*(*x*) in which *x* ∈ [*a*, *b*], and can examine its associated LPHVG with the limited penetrable distance *ρ*. Then

2 $$\begin{array}{c}P(k)\sim exp\{-(k-2\rho -\mathrm{2)}ln\mathrm{[(2}\rho +\mathrm{3)}/\mathrm{(2}\rho +\mathrm{2)]\}},\quad \rho =\mathrm{0,}\,\mathrm{1,}\,\mathrm{2,}\,\mathrm{...}\quad {\rm{with}}\quad \\ \quad \quad \quad k=2\rho +2,2\rho +\mathrm{3,}\ldots ,\forall f(x\mathrm{).}\end{array}$$

Note that when *ρ* = 0, then *P*(*k*) ~ *exp*[−(*k* − 2)*ln*(3/2)]^14^. For this case the LPHVG becomes the HVG (*Methods Section*). When *ρ* = 1, the result is Eq. (1). Therefore Eq. (2) is an extension of the previous result^14^ that indicates that the degree distribution *P*(*k*) of LPHVG associated with *i.i.d*. random time series has a unified exponential form.

**Figure 1:**
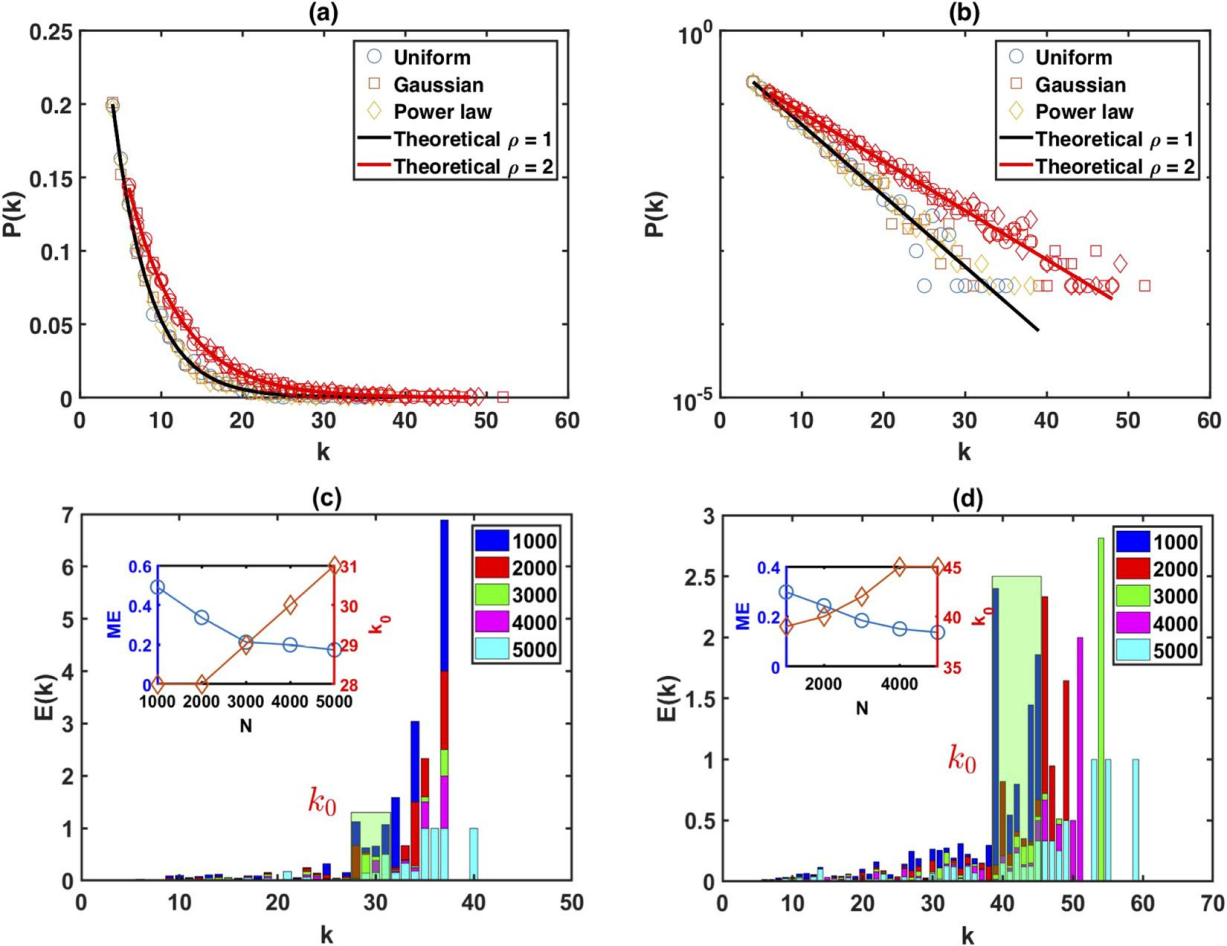
Numerical simulations of the degree distribution. (**a**) Degree distribution of the resulting graphs with penetrable distance *ρ* = 1 and *ρ* = 2, (**b**) semi-log plot of the degree distribution of the resulting graphs with penetrable distance *ρ* = 1 and *ρ* = 2, (**c**) test results of the resulting graphs with penetrable distance *ρ* = 1, (**d**) the test results of the resulting graphs with penetrable distance *ρ* = 2, both averaged over 10 realizations.

To further check the accuracy of our analytical results, we perform several numerical simulations. We generate a random series of 3000 data points from uniform, Gaussian, and power-law distributions and their associated limited penetrable horizontal visibility graphs. Figure 1(a) and (b) show plots of the degree distributions of the resulting graphs with a penetrable distance *ρ* = 1 and *ρ* = 2. Here circles indicate a series extracted from a uniform distribution, and squares and diamonds indicate series extracted from Gaussian and power-law distributions, respectively. The solid lines indicate the theoretical results of Eq. (2). We find that the theoretical results agree with the numerics. Note that one prerequisite for our theoretical results is that the length of the time series must be infinitely long, i.e. the series size *N* → ∞, so we can assert that the tail degree distribution of LPHVG associated to *i.i.d*. random series deviated from the theoretical result is only due to the effect of the finite size. In order to check the effect of the finite size, we define the relative error *E*(*k*) and the mean relative error *ME* to measure accurate between the numerical result under the finite size and the theoretical result. We also use a cutoff value *k*_0_ to denote the onset of finite size effects. Thus, we write

3 $$E(k)=\frac{|{P}_{num}(k)-{P}_{the}(k)|}{{P}_{the}(k)}\quad {\rm{and}}\quad ME=\sum _{k}E(k),$$

where, *P*_*num*_(*k*) and *P*_*the*_(*k*) denote the degree distribution of the numerical and theoretical results, respectively. We generate the random series from uniform distribution with different series sizes *N* and 10 realizations for each size. Figure 1(c) shows the test results of the resulting graphs with penetrable distance *ρ* = 1 and Fig. 1(d) with *ρ* = 2. The subplots in Fig. 1(c) and (d) show the relations between the mean relative error *ME* and the series size *N*, and the relations between the cutoff value *k*_0_ and the series size *N*. We find that when the finite size *N* increases, the cutoff value *k*_0_ also increases but the mean relative error *ME* decreases, which is in agreement with our previous assertion.

### Mean degree

Using Eq. (2) we calculate the mean degree 〈*k*〉 of the LPHVG associated with an uncorrelated random series,

4 $$\begin{array}{l}\langle k\rangle =\sum _{k}kP(k)=\sum _{k=\mathrm{2(}\rho +\mathrm{1)}}^{\infty }\frac{k}{2\rho +3}{(\frac{2\rho +2}{2\rho +3})}^{k-\mathrm{2(}\rho +\mathrm{1)}}=4(\rho +1).\end{array}$$

**Figure 2:**
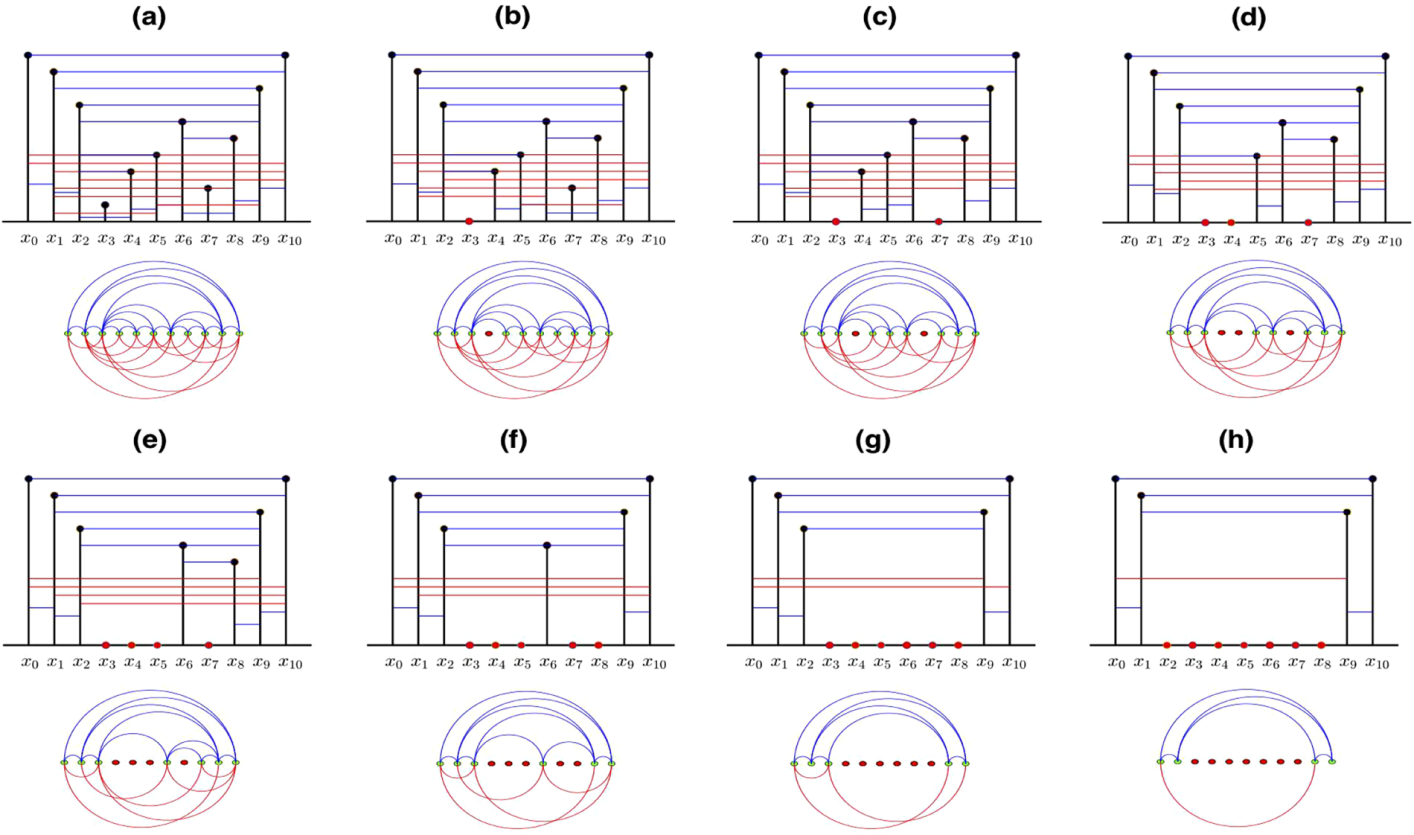
Graphical illustration of the constructive proof of 〈*k*(*T*)〉 considering a LPHVG with *ρ* = 1 extracted from a periodic series of period *T* = 10.

Next, we deduce a more general expression of mean degree 〈*k*(*T*)〉, which depends on the period of a time series *T*. We consider an infinite periodic series of period *T* denoted by *X*_*t*_ = {..., *x*_0_, *x*_1_, *x*_2_, ..., *x*_*T*_, *x*_1_, *x*_2_, ...}, where *x*_0_ = *x*_*T*_ and no repeated values in a period. Let *ρ* ≪ *T* for the subseries ${\tilde{X}}_{t}=\{{x}_{0},{x}_{1},{x}_{2},\,\mathrm{...,}\,{x}_{T}\}$. Without loss of generality, we assume *x*_0_ = *x*_*T*_ corresponds to the largest value of the subseries, and *x*_1_, ..., *x*_*ρ*_, *x*_*T*−*ρ*_, ...*x*_*T*−1_ corresponds to the (2*ρ* + 1)nd largest value of the subseries. We then can construct the LPHVG associated with the subseries ${\tilde{X}}_{t}$. If the LPHVG has *E* links and *x*_*i*_ is smallest datum of ${\tilde{X}}_{t}$, the degree of *x*_*i*_ is 2(*ρ* + 1) during the construction of LPHVG. Figure S1 illustrates the result for *ρ* = 1. We delete node *x*_*i*_ and its 2(*ρ* + 1) links from the LPHVG and the resulting graph has *E* − 2(*ρ* + 1) links and *T* nodes. We iterate this process *T* − (2*ρ* + 1) times and in Fig. 2 we present a graphical illustration for *ρ* = 1 and *T* = 10. The total number of deleted links is now *E*_*d*_ = 2(*ρ* + 1)[*T* − (2*ρ* + 1)] and the resulting graph has 2(*ρ* + 1) nodes, i.e., *x*_0_, *x*_1_, ..., *x*_*ρ*_, *x*_*T*−*ρ*_, ...*x*_*T*−1_, *x*_*T*_, as shown in Fig. 2(h) for *ρ* = 1 and *T* = 10. Because these 2(*ρ* + 1) nodes are connected by $${E}_{r}=(\begin{array}{c}\mathrm{2(}\rho +\mathrm{1)}\\ 2\end{array})$$ links, the mean degree of a limited penetrable horizontal visibility graph associated with *X*_*t*_ is

5 $$\begin{array}{rcl}\langle k(T)\rangle =2(\frac{{E}_{d}+{E}_{r}}{T}) & = & \frac{2[\mathrm{2(}\rho +\mathrm{1)(}T-\mathrm{(2}\rho +\mathrm{1))}+(\rho +\mathrm{1)(2}\rho +\mathrm{1)}]}{T}\\  & = & 4(\rho +1)(1-\frac{2\rho +1}{2T}),\,\rho \ll T\mathrm{.}\end{array}$$

Note that Eq. (5) holds for every periodic or aperiodic series in which *T* → ∞. This is independent of the deterministic process that generates the series, since the only constraint in its derivation is that no data repeatitions in a period is allowed. Note that one consequence of Eq. (5) is that every time series has an associated LPHVG with the maximum mean degree (achieved for aperiodic series) 〈*k*(∞)〉 = 4(*ρ* + 1), which agrees with Eq. (4).

**Figure 3:**
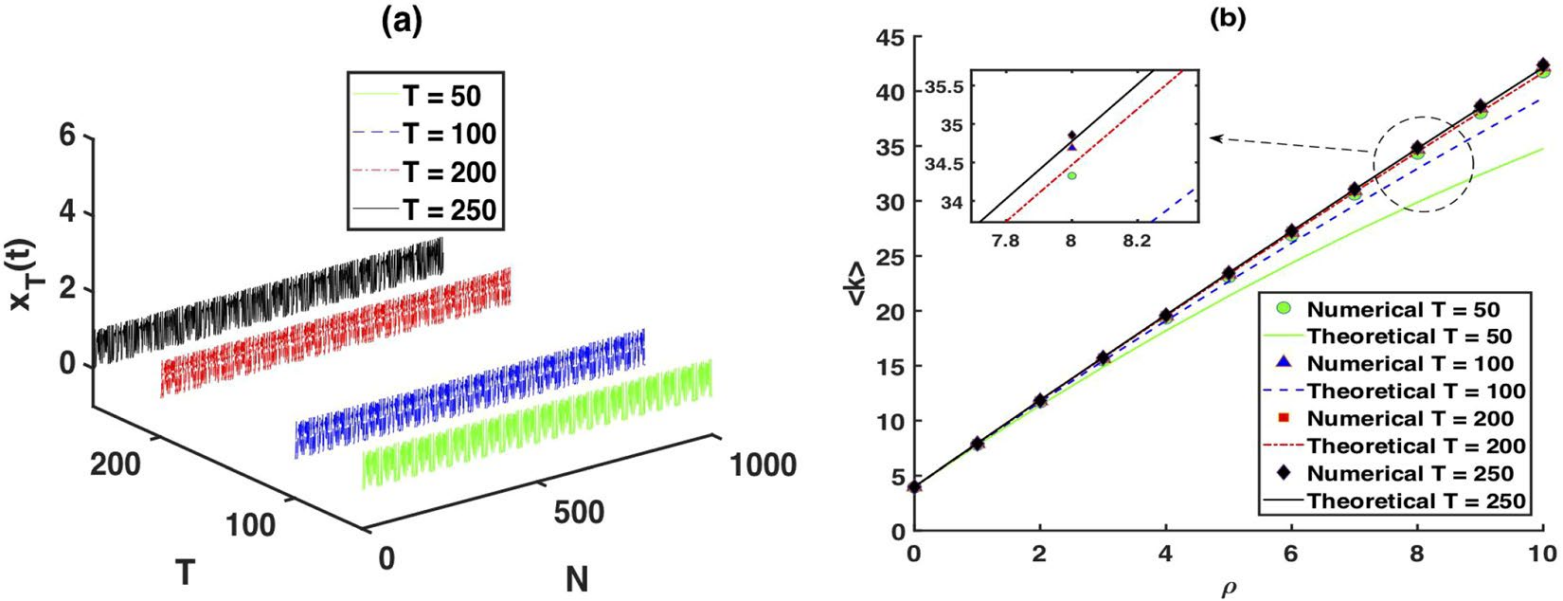
Numerical simulations of mean degree. (**a**) A simplified period-50, period-100, period-200 and period-250 time series of 1000 data. (**b**) The mean degree of the resulting LPHVGs with different *ρ*, where the time series with periodic-50, periodic-100, periodic-200 and periodic-250 are represented by circles, triangles, squares and diamonds, respectively. The solid lines correspond to the theoretical result for each time series.

To check the accuracy of our analytical result, we generate simple period-50, period-100, period-200, and period-250 time series with 1000 data points, as illustrated in Fig. 3(a). We construct the limited penetrable horizontal visibility graphs with the penetrable distance *ρ* = 0, 1, 2, ..., 10 associated with this periodic time series. Figure 3(b) is a plot of the mean degree of the resulting LPHVGs with different *ρ* values, where we found a good agreement with the numeric results for *ρ* ≪ *T*.

### Local clustering coefficient

In LPHVG, nodes with the same degree usually have different clustering coefficients since the degree of the node contribute with different configurations and structures (see proof of Theorem S1). By calculating these coefficients for different configurations (Theorem S3), we find that they are irregular, but the minimum and the maximum clustering coefficient for nodes in LPHVG are regular. Therefore, based on results of degree distribution [Eq. (2)], we can deduce the minimum and maximum local clustering coefficient *C*_*min*_(*k*) and *C*_*max*_(*k*) of the LPHVG associated to *i.i.d*. random series. Thus, we write

6 $${C}_{{\rm{\min }}}(k)=\frac{2}{k}+\frac{2\rho (k-\mathrm{2)}}{k(k-\mathrm{1)}},\quad \rho =0,1,2\quad {\rm{and}}\quad k\ge \mathrm{2(}\rho +\mathrm{1).}$$

7 $${C}_{{\rm{\max }}}(k)=\frac{2}{k}+\frac{4\rho (k-\mathrm{3)}}{k(k-\mathrm{1)}},\quad \rho =0,1,2\quad {\rm{and}}\quad k\ge \mathrm{2(2}\rho +\mathrm{1).}$$

Using Eqs (2, 6) and (7) we also obtain the local clustering coefficient distribution *P*(*C*_min_) and *P*(*C*_max_), i.e.,

8 $$\begin{array}{rcl}P({C}_{{\rm{\min }}}) & = & \frac{1}{2\rho +3}exp\{[\frac{\phi +\sqrt{{\phi }^{2}-8{C}_{{\rm{\min }}}\mathrm{(2}\rho +\mathrm{1)}}}{2{C}_{{\rm{\min }}}}-\mathrm{2(}\rho +\mathrm{1)}]ln(\frac{2\rho +2}{2\rho +3})\},\\ \phi  & = & {C}_{{\rm{\min }}}+2\rho +\mathrm{2,}\end{array}$$

9 $$\begin{array}{rcl}P({C}_{{\rm{\max }}}) & = & \frac{1}{2\rho +3}exp\{[\frac{\varphi +\sqrt{{\varphi }^{2}-8{C}_{{\rm{\max }}}\mathrm{(6}\rho +\mathrm{1)}}}{2{C}_{{\rm{\max }}}}-\mathrm{2(}\rho +\mathrm{1)}]ln(\frac{2\rho +2}{2\rho +3})\},\\ \varphi  & = & {C}_{{\rm{\max }}}+4\rho +2.\end{array}$$

**Figure 4:**
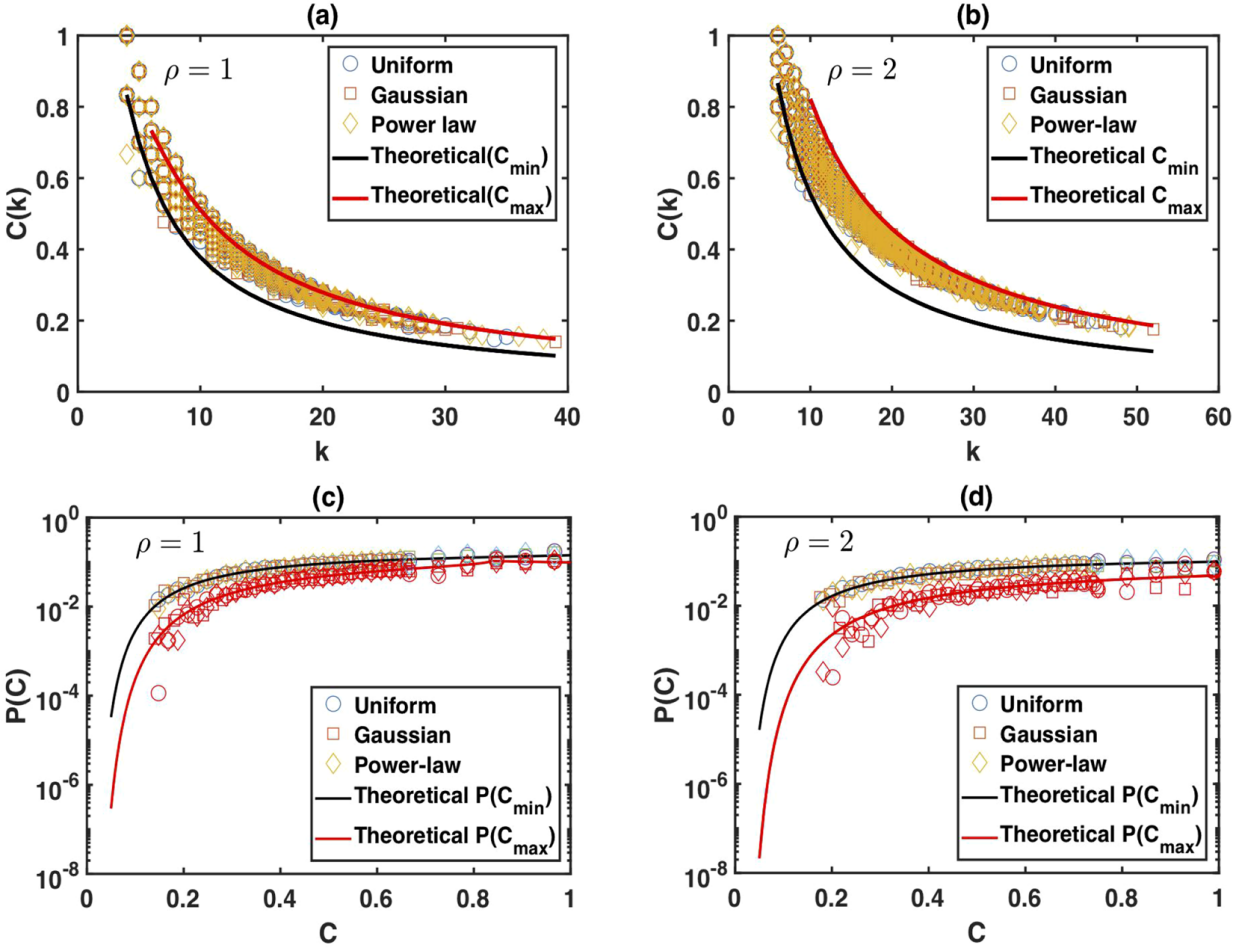
Clustering coefficient of LPHVG versus degree of the nodes for (**a**) *ρ* = 1 and (**b**) *ρ* = 2. The theoretical prediction of *C*_min_(*k*) is shown in a solid black line and a red line denotes the prediction for *C*_max_(*k*). In (**c**) and (**d**) we plot the clustering coefficient distribution and the theoretical prediction lines for *ρ* = 1 and *ρ* = 2, respectively.

For a proof of this result see Theorem S3 in the Supplementary Information. Figure 4 shows the clustering coefficient *C*(*k*) and its distribution *P*(*C*) for limited penetrable horizontal visibility graphs associated with different random series of 3000 data points. The solid black and red lines in Fig. 4(a) and (b) are the theoretical prediction of *C*_min_(*k*) and *C*_max_(*k*), respectively [see Eqs (6) and (7)]. In Fig. 4(c) and (d) we show a similar analysis for *P*(*C*_min_) and *P*(*C*_max_) [see Eqs (8) and (9)].

Figure 4 shows that the theoretical predictions of *C*_min_(*k*), *C*_max_(*k*), *P*(*C*_min_), and *P*(*C*_max_) agree with the numerics.

### Long distance visibility

Consider a limited penetrable horizontal visibility graph associated with a bi-finite sequence of *i.i.d*. random variables, which are extracted from a continuous probability density *f*(*x*). The probability *P*_*ρ*_(*n*) that two data points are connected, when they are separated by *n* intermediate data points, is given by

10 $${P}_{\rho }(n)=\frac{2\rho (\rho +\mathrm{1)}+2}{n(n+\mathrm{1)}}\mathrm{.}$$

**Figure 5:**
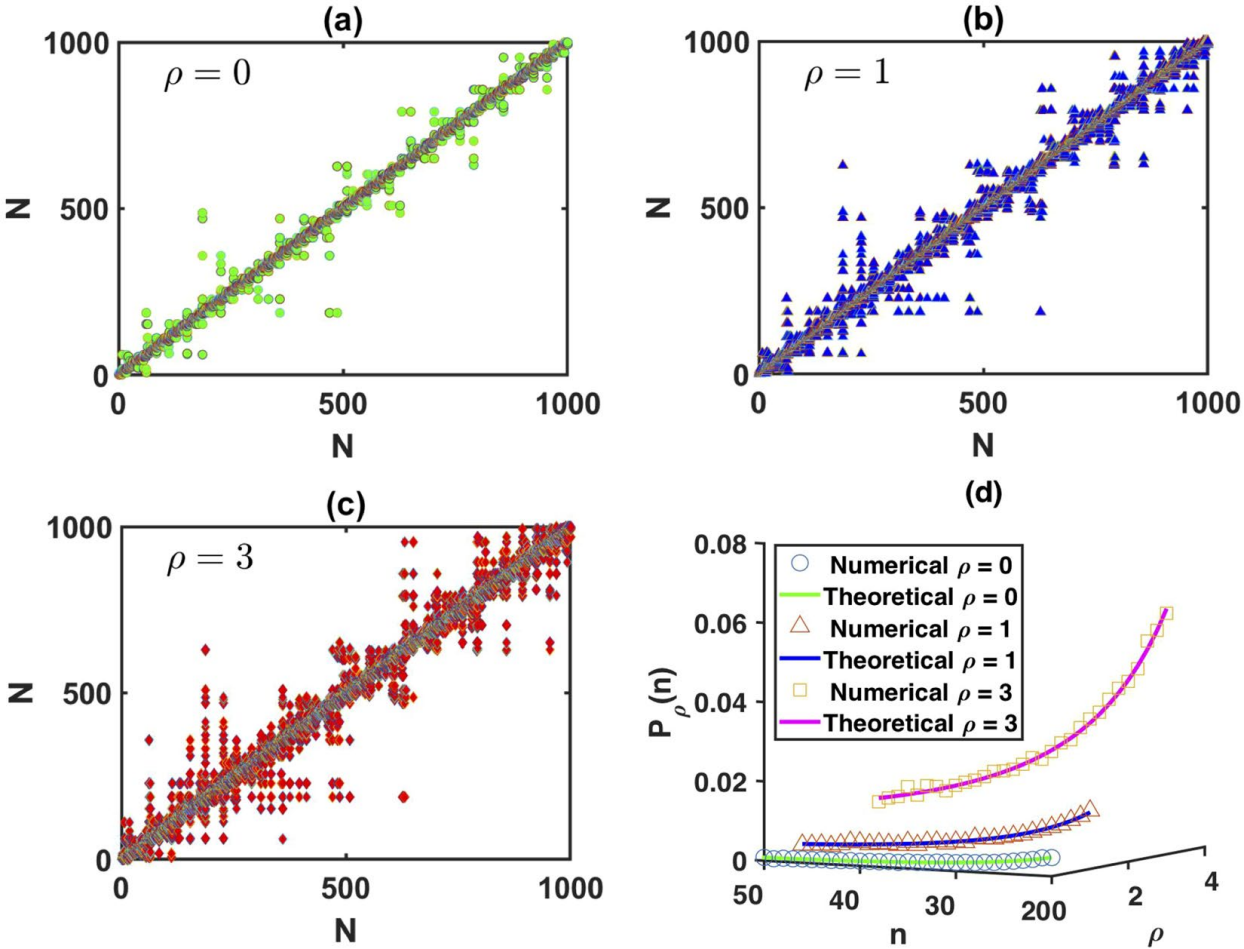
Adjacency matrix of LPHVGs associated to a random series for (**a**) *ρ* = 0, (**b**) *ρ* = 1 and (**c**) *ρ* = 3. (**d**) Plot of the dependence of P_*ρ*_(*n*) with *ρ* and *n* (the solid line corresponds to the theoretical result [Eq. (10)]. Circles, triangles and diamonds correspond to the result of numerical simulations for *ρ* = 0, *ρ* = 1 and *ρ* = 3, respectively.

Note that *P*_*ρ*_(*n*) is again independent of the probability distribution of the random variable *x*. For a detailed proof of this result see Theorem S4 in the Supplementary Information. Figure 5(a,b) and (c) show the adjacency matrix *A* of the limited penetrable horizontal visibility graph associated with a random series for different limited penetrable distances. When *A*(*i*, *j*) = 1, we plot *ρ* = 0 (circles), *ρ* = 1 (triangles), and *ρ* = 3 (diamonds) at each element of the matrix.

As we observed, Fig. 5(a,b) and (c) shows a typical homogeneous structure in which the adjacency matrix is filled around the main diagonal. In addition, the matrix indicates a superposed sparse structure caused by the limited penetrable visibility probability *P*_*ρ*_(*n*), introducing shortcuts into the limited penetrable horizontal visibility graph. These shortcuts indicate that the limited penetrable horizontal visibility graph present small-world features. Figure 5(b) shows that the theoretical result in Eq. (10) agrees with the numerics and these results are exact, with regard to the topological properties of the LPHVG associated with *i.i.d*. random series.

### Application to deterministic chaotic time series

These results can be used to discriminate between random and chaotic signals. Methods to analyze random processes and identify its behavior as chaotic or stochastic has received extensive study in recent decades^38–41^. Most previous algorithms have been phenomenological and computationally complicated. Thus, new methods that can reliably distinguish stochastic from chaotic time series are needed. Recently Lacasa *et al*.^13,14^ used the horizontal visibility algorithm to characterize and distinguish between chaotic and stochastic processes. Here we use our new theory to distinguish chaotic series from random series and compare with the horizontal visibility algorithm^13,14^.

We address four deterministic time series. First, generated by the Logistic map^42^, we have

$${x}_{t+1}=\mu {x}_{t}\mathrm{(1}-{x}_{t})\quad {\rm{with}}\quad \mu =4.$$

Second, the Hénon map^43^

$${x}_{t+1}=1+{y}_{t}-a{x}_{t}^{2},\quad {y}_{t+1}=b{x}_{t}\quad {\rm{with}}\quad a=1.4\quad {\rm{and}}\quad b=\mathrm{0.3.}$$

The Lorenz chaos system^44^,

$$\dot{x}=a(y-x),\quad \dot{y}=cx-y-xz,\quad \dot{z}=xy-bz\quad {\rm{with}}\quad a=10\quad {\rm{and}}\quad b=8/3,\quad {\rm{and}}\quad c=28;$$

and last, the energy price-supply-economic growth system^34^,

$$\dot{x}={a}_{1}x+{a}_{2}(C-y)+{a}_{3}(z-{K}_{1}),\quad \dot{y}=-{b}_{1}\,y+{b}_{2}x-{b}_{3}z\mathrm{(1}-z/{K}_{2}),\quad \dot{z}={c}_{1}z\mathrm{(1}-z/L)+{C}_{2}\,yz,$$

where *a*_1_ = 0.3, *C* = 27, *a*_2_ = 0.5563, *a*_3_ = 0.15, *b*_1_ = 0.4, *b*_2_ = 0.6073, *b*_3_ = 0.3, *K*_1_ = 15, *K*_2_ = 15, *c*_1_ = 0.3, *c*_2_ = 0.006 and *L* = 19.

**Figure 6:**
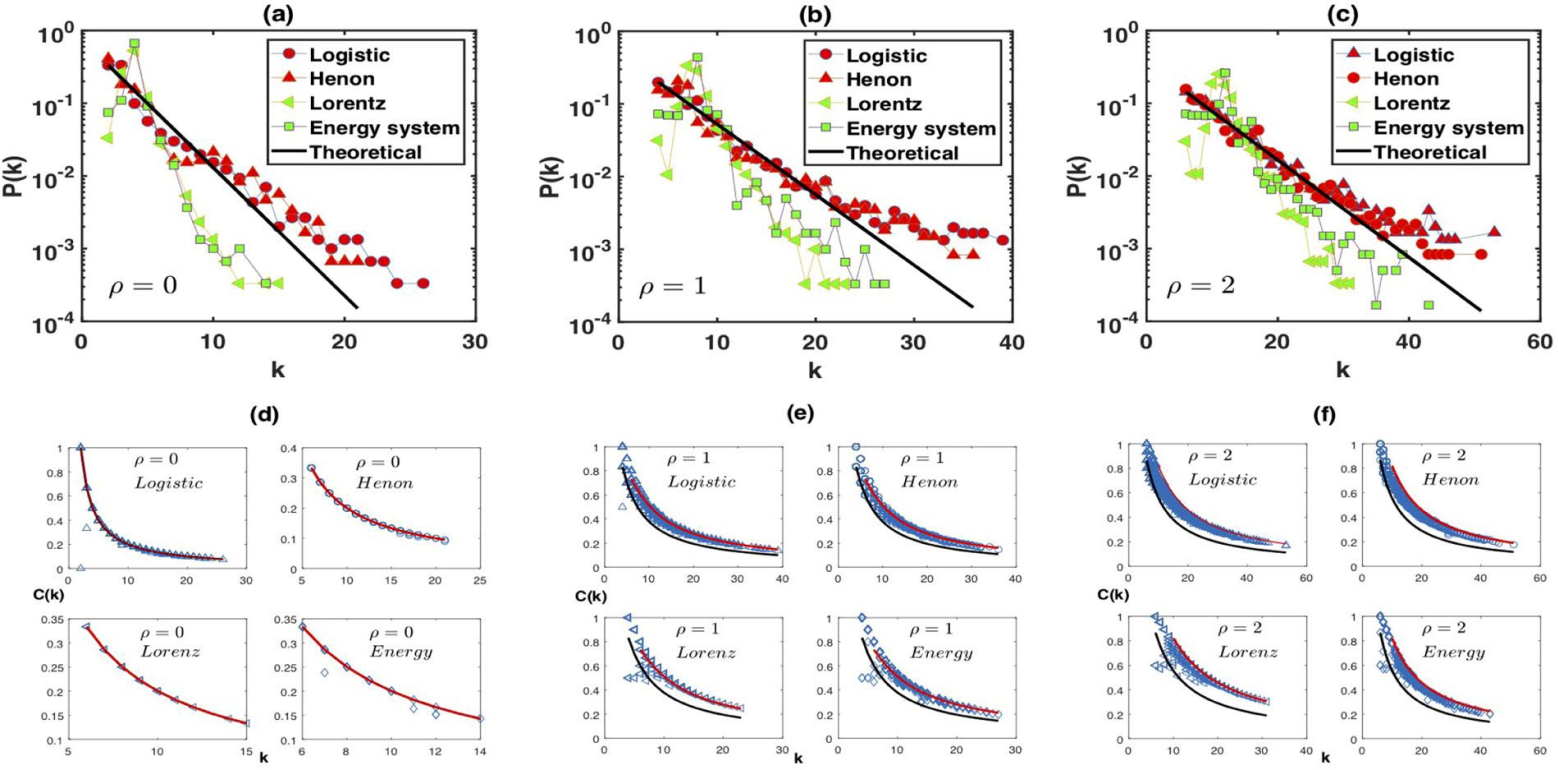
Numerical results for different chaotic series with semilog plots for the degree distributions of LPHVGs associated to series generated by Logistic map, Hénon map, Lorenz chaotic system and Energy price-supply-economic growth system. In (**a**) we have *ρ* = 0, (**b**) *ρ* = 1 and (**c**) *ρ* = 2. In bottom panels we show the relation between degree and clustering coefficient for (**d**) *ρ* = 0, (**e**) *ρ* = 1 and (**f**) *ρ* = 2. The solid black and red lines in these latter panels are the theoretical prediction of *C*_min_(*k*) and *C*_max_(*k*), respectively.

Figure 6 shows the limited penetrable horizontal visibility graphs of 3000 data points extracted from two different chaotic maps and two different chaotic systems. Here, we show our results for the degree distribuction calculated numerically for (a) *ρ* = 0, (b) *ρ* = 1 and (c) *ρ* = 2. In the bottom panel, we plot the relation between clustering coefficient and degrees of the nodes. In every case *P*(*k*) deviates from Eq. (2) and *C*(*k*) deviates from Eqs (6) and (7). We also find that the degree distributions of the LPHVGs associated with these chaotic maps and systems can be approximated using the exponential function $P(k)\sim exp(-\hat{\lambda })k$, but $\hat{\lambda }\ne \lambda =ln[\mathrm{(2}\rho +\mathrm{3)}/\mathrm{(2}\rho +\mathrm{2)}]$ in each case. We conjecture that there is a functional relation between the random and chaos dimensions^13,14^. Thus the parameter *λ* is the frontier between random series and chaotic series and can be used to distinguish randomness from chaos. The solid black and red lines in the bottom panel of Fig. 6 are the theoretical prediction of *C*_min_(*k*) and *C*_max_(*k*), respectively [see Eqs (6) and (7)]. In particular, both lines collapse in Fig. 6(d). This result shows that the limited penetrable horizontal visibility graphs for (e) *ρ* = 1 and (f) *ρ* = 2 are better discriminators from the perspective of *C*(*k*) than the horizontal visibility graph of *ρ* = 0 shown in Fig. 6(d).

### Application to real crude oil future price series

**Figure 7:**
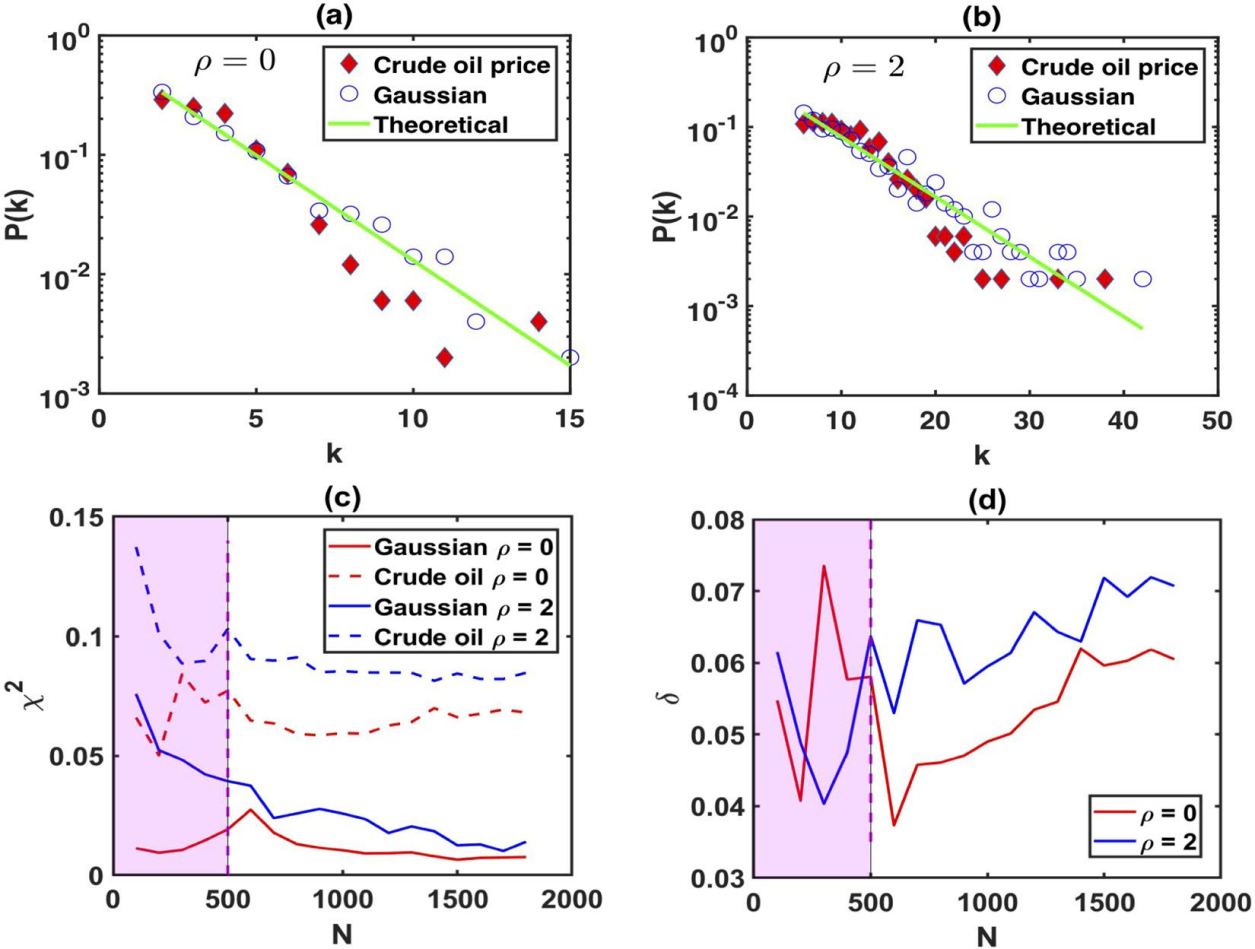
Semilog plot of the degree distributions for (**a**) HVG associated to crude oil price and Gaussian random series and (**b**) for the LPHVG with *ρ* = 1. (**c**) Plot of the numerical values of *χ*^2^ of LPHVGs with *ρ* = 0 and *ρ* = 2 associated to crude oil price and Gaussian random series. (**d**) Plot for numerical values of *δ* of LPHVGs with *ρ* = 0 *ρ* = 2 for different data sizes.

As a further example, we use the data from the U.S. Energy Information Administration on the crude oil future contract 1 (Dollars per Barrel) from April 4, 1983 to March 28, 1985, and we found that they exhibit chaotic and long-range correlations^45,46^. We select 500 sample data points and demonstrate that we can use our method to distinguish chaotic series from random series when the data sample is small (although for theoretical results we need infinite data). Figure 7 shows the results of LPHVG with *ρ* = 0, i.e. HVG [see Fig. 7(a)] and LPHVG with *ρ* = 2 [see Fig. 7(b)] of 500 data points extracted from Gaussian random series and crude oil futures. Here circles indicate a series extracted from a Gaussian distribution and diamonds indicate series extracted from crude oil futures. The solid green line is the theoretical value of *P*(*k*) given by Eq. (2). We can find that the degree distributions associated with Gaussian random series and crude oil price series both deviate from Eq. (2). From the above analysis, the deviations between the tails of the degree distributions associated with Gaussian random series are caused solely by finite size effects. However, how the degree distributions associated with crude oil price deviate from Gaussian random series is not clear. To quantify the deviations of these two cases, we compute the quantity *χ*^2^

11 $$\begin{array}{l}{\chi }^{2}=\sum _{k}\frac{{[{P}_{{\rm{num}}}(k)-{P}_{{\rm{theo}}}(k)]}^{2}}{{P}_{{\rm{theo}}}(k)},\end{array}$$

where *P*_num_(*k*) is the degree distribution of the numerical result and *P*_theo_(*k*) the theoretical result from Eq. (2). Note that *χ*^2^ changes with the sample size *N*. Figure 7(c) shows a plot of the numerical values of *χ*^2^ of LPHVGs with *ρ* = 0 and *ρ* = 2 associated with Gaussian random series and crude oil price series for different data sizes. From Fig. 7(c) we can clearly see that the degree distributions associated with crude oil price deviate from Gaussian random series, which means the crude oil future price sequence is not random but chaotic. Furthermore, we compute the absolute distance *δ*

12 $$\begin{array}{l}\delta =|{\chi }_{Gauss}^{2}-{\chi }_{oil}^{2}|,\end{array}$$

where ${\chi }_{Gauss}^{2}$ and ${\chi }_{oil}^{2}$ are *χ*^2^ value for Gaussian random series and the value of crude oil price series, respectively. The larger *δ* means a better distinction between the series. Figure 7(d) shows a plot of the numerical values of *δ* of LPHVGs with *ρ* = 0 and *ρ* = 2 of different data size. Comparing the results of HVG (*ρ* = 0) and LPHVG (*ρ* = 2), we find that when *N* ≥ 500 LPHVG works better than HVG to distinguish the real time series from uncorrelated randomness. Therefore, in the next section, we select 500 sample data in each sliding window, i.e. *L* = 500, to construct LPHVG for measuring the global evolution characteristics of crude oil price series.

**Figure 8:**
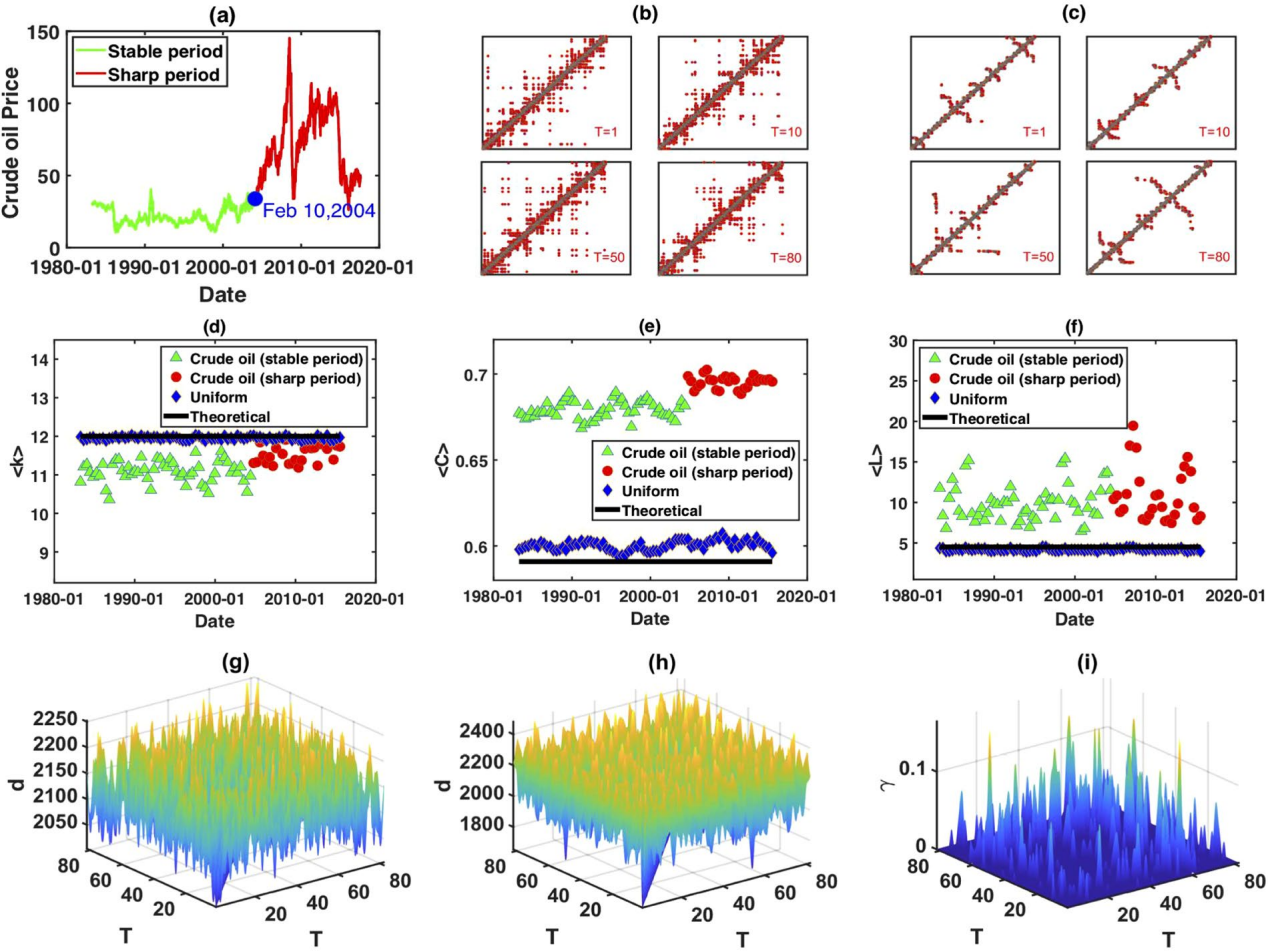
(**a**) Crude oil price series, where the green line represents the stable period and the red line represents the sharp period. (**b**) evolution of the adjacency matrix of LPHVGs associated to the random series extracted from a uniform distribution. (**c**) Evolution of the adjacency matrix of LPHVGs associated to crude oil price series. (**d**) Mean degree of the LPHVGs associated to random series, crude oil price series and the predicted theoretical value. (**e**) Mean clustering coefficient of the LPHVGs associated to random series, crude oil price series and the theoretical value. (**f**) Mean path length of the LPHVGs associated to random series, crude oil price series and the theoretical value. (**g**) Distance distribution of random series. (**h**) Distance distribution of crude oil price series. (**i**) Correlation index distribution of crude oil price series.

Next we use LPVHG with *ρ* = 2 to describe the global evolution of crude oil future prices (for the calculation process, see Methods). Our sample data is from the crude oil future contract 1 (in dollars per barrel) from April 4, 1983 to August 15, 2017 [Fig. 8(a)]. As we know, the fluctuations in crude oil future prices differ for several time periods^24,47^. The whole crude oil price time series can be divided into five different fluctuation periods, based on complex network perspective: stable fluctuation period (April 4, 1983 to February 10, 2004), sharp rise period (February 11, 2004 to June 30, 2007), sharp decline period (July 1, 2007 to November 20, 2008), sharp rise period (November 20, 2008 to June 19, 2013) and sharp decline period (June 20, 2013 to August 15, 2017). Here, instead of discussing the fluctuation characteristics of each period, we want to show that the fluctuation behavior in different periods can be revealed by the topological structure of LPHVG. Thus, for simplicity, we combine the sharp rise and sharp decline periods into one sharp fluctuation period and we only consider two periods: a stable period from April 4, 1983 to 10 February 2004 and a period of sharp fluctuations from 11 February 2004 to 15 August 2017. Using our calculation method (see the Method section), we establish 82 windows where each window has 100 weeks in the time series (i.e., *L* = 500), the first of which is from 4 April 1983 to 28 March 1985. Because each window moves 20 weeks to generate the next window (i.e., *l* = 100), two adjacent windows have overlaps of 80 weeks. This enables information from one window to move to the next in succession. Each window contributes with 500 nodes to build the local limited penetrable visibility graph network. Figure 8(b) and (c) show the evolution of the adjacency matrix of LPHVGs associated with a random series extracted from a uniform distribution and from crude oil price, respectively. Note that adjacent matrices in the random time series and the crude oil price time series significantly differ, but their respective adjacent matrices in different time windows are similar. Figure 8(d–f) show the evolution of the mean degree, mean clustering coefficient and mean path length, respectively. We find that the mean degree, mean clustering coefficient and mean path length of the LPHVG associated with the random series agree with the theoretical values, but these three quantities of LPHVG associated with the crude oil price series do not. The levels of mean degree of the LPHVG associated with the crude oil price series are smaller than the theoretical values, but the mean clustering coefficient and mean path length are larger. They also show different trends in different fluctuation periods. All the quantities plotted present larger values in the sharp fluctuation period than in the stable fluctuation period. Figure 8(g) and (h) show the distance distribution for a random and for crude oil price series (see Eqs (14–15) in Methods). Comparing the results of Fig. 8(g) and (h), we find that the range of distance of crude oil price series is larger than random series, which means the correlation of crude oil price fluctuation presents high complexity. Based on the result of Fig. 8(g) and Eq. (16), we can determine the threshold value *θ* = 2001, then using Eq. (17), we can obtain the correlation index distribution of crude oil price series. Note that the *i*.*i*.*d*. random time series has neither short-range nor long-range correlations, but the crude oil price time series has both. Thus using LPHVG enables us to describe the global time series evolution.

## Discussion

We have introduced a more generalized case of the horizontal visibility algorithm^13,14^, named limited penetrable horizontal visibility algorithm, in which the limited penetrable distance is *ρ*. We obtain exact results on several properties of the limited penetrable horizontal visibility graph associated with a general uncorrelated random series, and its reliability has been confirmed by numerical simulations. In particular, the degree distribution of the graph has the exponential form and the expression for the mean degree holds for every periodic or aperiodic series, which does not depend on the deterministic process that generates them. We also found that the clustering coefficient *C* has a maximum and a minimum value, which depend on the degree *k* and distance *ρ*. We concluded that the probability ${P}_{\rho }(n)=\frac{2\rho (\rho +1)+2}{n(n+1)}$ introduces shortcuts to the limited penetrable horizontal visibility graph that exhibit a small-world phenomenon. These results are independent of the distribution from which the series was generated and we observe that all uncorrelated random series have the same limited penetrable horizontal visibility graph and, in particular, the same degree distribution, mean degree, clustering coefficient distribution, and small-world characteristics.

**Figure 9:**
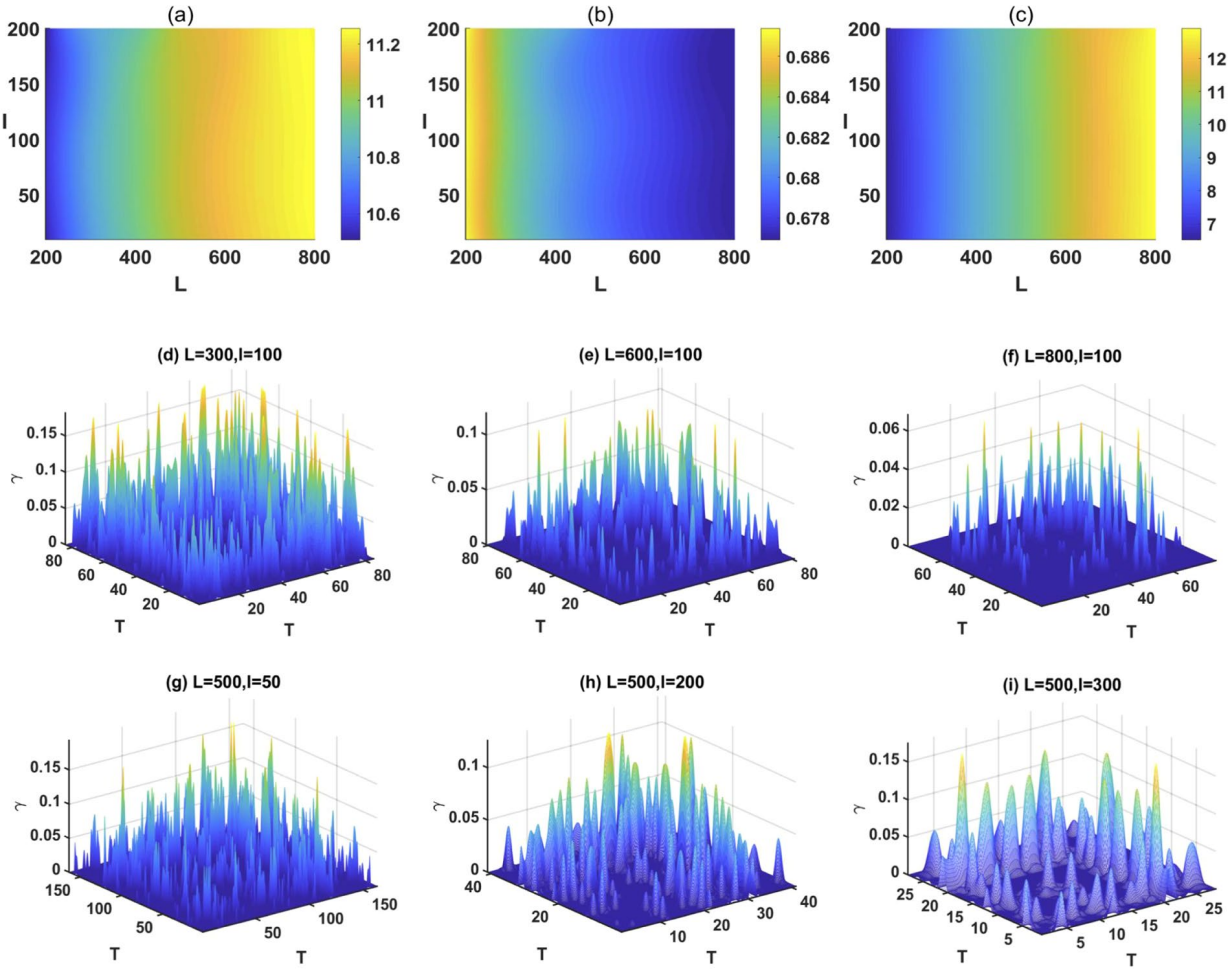
Sensitivity analysis on *L* and *l* for crude oil price series. In (**a**) we show the mean degree, (**b**) the mean clustering coefficient, (**c**) the mean path length. In (**d**–**i**) we show th correlation index distributions for different values of scale *L* and *l*.

This algorithm can thus be used as a simple test for discriminating uncorrelated randomness from chaos. We show that the method can distinguish between random series that follow the theoretical predictions, and chaotic series that deviate from them. In addition, we employ the method to measure the global evolution characteristics of time series by using LPHVG with *ρ* = 2, and the results confirmed its validity. Note that we set *L* = 500, *l* = 100 as an example to construct the continuous LPHVGs in this paper, moreover, we can set different scales of sliding window *L* and step length *l* depending on the needs of the analysis. If the goal is to study the global evolution characteristics based on short periods, scales *L* and *l* can be set to smaller values. On the contrary, the scales *L* and *l* can be set to larger values. Increasing th scale *L* and *l*, the mean degree and mean path length in the corresponding complex networks increase, as shown in Fig. 9(a) and (c) and the mean clustering coefficient decreases [Fig. 9(b)]. The parameters *L* and *l* determine the number of sliding windows, and for crude oil price time series, when *L* = 500, *l* = 100 the number of sliding windows is 82. This number is only 28 if *L* = 500, *l* = 300. We observe that an increase in scale *L* and *l* will hide the characteristics of diversity of the real time series. On the other hand, for *L* = 50, *l* = 10 the number of sliding windows is more than 800, and they contain much noise. Thus, if the values of scale *L* and *l* are too large or too small, it is meaningless for studying the global evolution characteristics of the real time series. When we determine the appropriate parameters *L* and *l*, the corresponding threshold *θ* can be calculate by using Eqs (14–16) and we can observe the similarly distribution characteristics of correlation index of crude oil price time series, as illustrated in Fig. 9(d–i).

Our exact results presented here are extension of previous work^14^ and the method can serve as a preliminary test for locating deterministic fingerprints in time series. To better distinguish chaos from uncorrelated randomness, the limited penetrable parameter *ρ* can be adjusted. If we determine that *P*(*k*) has an exponential tail that deviates from the theoretical values or that *C*(*k*) also deviates from the theoretical results [Eqs (2, 6) and (7)], we apply embedding methods to analyze the series. There are also some open problems unsolved in this work, and the main question is how to deduce the theoretical results of LPHVG associated with more complicated time series, such as logistic maps, fractional Brownian motions and Lorenz equations. Another question is how to use this algorithm to measure chaos and reveal the global evolution characteristics for other time series. Therefore, topics for further research could include whether this algorithm is also able to quantify chaos, the relation between such standard chaos indicators as Lyapunov exponents and the correlation dimension. Also, how to tune the limited penetrable parameter *ρ* and how to use the limited penetrable horizontal visibility graph to handle two-dimensional manifolds. The topological properties of the visibility graphs (VG), of the limited penetrable visibility graphs (LPVG) and expanded applications of LPHVG might be considered in future works.

## Methods

### Limited Penetrable Horizontal Visibility Graph [LPHVG]

**Figure 10:**
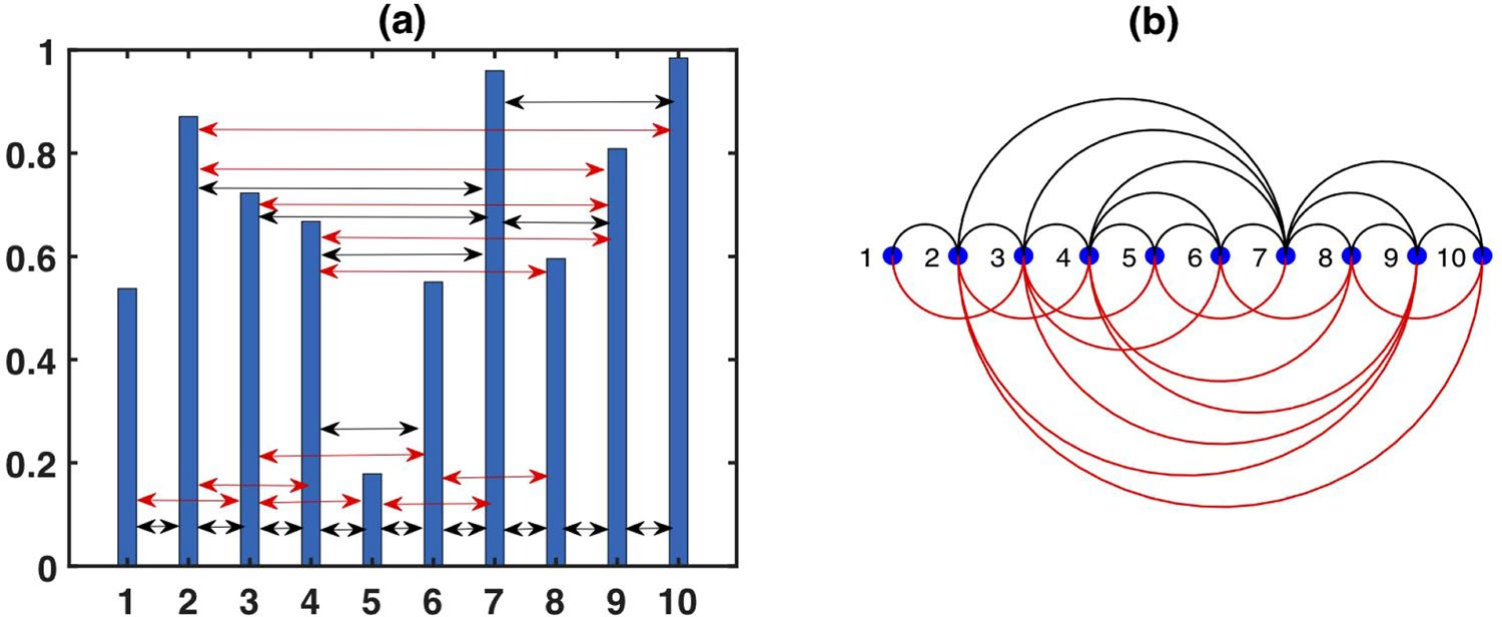
Example of (**a**) a time series with 10 data values and (**b**) its corresponding LPHVG with the limited penetrable distance *ρ* = 1, where every node corresponds to a time series data. The limited penetrable horizontal visibility lines between data points define the links connecting nodes in the graph.

The limited penetrable visibility graph (LPVG)^37^ and the multiscale limited penetrable horizontal visibility graph (MLPHVG)^17^ are a recent extension of the VG^12^ and HVG^13,14^ used to analyze nonlinear time series. The limited penetrable horizontal visibility graph (LPHVG) is geometrically more simple and an analytically solvable version of LPVG^37^ and MLPHVG^17^. To define it we let *X*_*t*_ = {*x*_*i*_}, *i* = 1, 2, ..., *N* be a time series of *N* real numbers. If we set the limited penetrable distance to *ρ*, LPHVG maps the time series into a graph with *N* nodes and an adjacency matrix *A*. Nodes *i* and *j* are connected through an undirected edge (*A*_*ij*_ = *A*_*ji*_ = 1) when *x*_*i*_ and *x*_*j*_ have limited penetrable horizontal visibility (see Fig. 10), i.e., if at most *ρ*, the intermediate data *x*_*q*_ is

13 $${x}_{q}\ge inf\{{x}_{i},{x}_{j}\},\forall q\in (i,j),$$

this mapping is a limited penetrable horizontal visibility graph (LPHVG). When we set the limited penetrable distance *ρ* = 0, LPHVG degenerates into HVG^14^. When *ρ* ≠ 0, there are more connections between any two nodes in LPHVG than in HVG. Figure 10(b) shows the new established connections (red lines) when we infer the LPHVG on the basis of HVG with a limited penetrable distance *ρ* = 1. Note that the limited penetrable horizontal visibility graph of a given time series has all the properties of its horizontal visibility graph, e.g., it is connected and invariant under all affine transformations of the series data^12,14^.

### Measurement of the Global Evolution Characteristics of Time Series using LPHVG

To characterize the evolution of the time series *X*_*t*_ = {*x*_*i*_}, *i* = 1, 2, ..., *N* using LPHVG, we divide the entire scale of the time series into equal small-scale segments (or windows) and assume that the length of the sliding window is *L*. We define *l* as the step length between sliding time windows. To ensure that small-scale segments of the time series are continuous, we require that *l* < *L*. This allows us to obtain *T* = [(*N* − *L*)/*l* + 1] for small-scale time windows, where [...] is the rounding function. For every small-scale time window *t*, we transform time series into the a LPHVG of time *t* using the limited penetrable horizontal visibility algorithm. The topological structure of LPHVG changes with time *t*, therefore, we write *LPHVG*(*t*). In order to describe this process, we use the Euclidean distance to measure the relationship between LPHVGs. We define the Euclidean distance between *LPHVG*(*t*_*m*_) and *LPHVG*(*t*_*n*_) to be

14 $$d[LPHVG({t}_{m}),LPHVG({t}_{n})]=\sqrt{\sum _{i=1}^{L}\sum _{j=1}^{L}({a}_{ij}^{({t}_{m})}-{a}_{ij}^{({t}_{n})})},\quad {\rm{where}}\quad {a}_{ij}^{({t}_{m})}\in {{\rm{A}}}^{{t}_{m}}\quad {\rm{and}}\quad {a}_{ij}^{{t}_{n}}\in {{\rm{A}}}^{{t}_{n}}\mathrm{.}$$

We then determine the distance matrix

15 $${D}_{T\times T}={\{{d}_{{t}_{m},{t}_{n}}\}}_{{t}_{m}=1,\mathrm{2,...,}T,{t}_{n}\mathrm{=1,2,...,}T},$$

and assign a threshold value to *θ*

16 $$\theta =min{\{{d}_{tm,{t}_{n}}^{{\rm{rand}}}\}}_{{t}_{m}\ne {t}_{n}},\quad {d}_{{t}_{m},{t}_{n}}^{{\rm{rand}}}\in {{D}}_{T\times T}^{{\rm{rand}}}\mathrm{.}$$

Here ${{D}}_{T\times T}^{{\rm{rand}}}$ is the distance matrix associated with the independent and identically distributed random time series. From Eq. (16), we can see that *θ* can be obtained from the distance matrix of LPHVGs associated with the *i.i.d*. time series, which is the critical value to measure the correlation between the data in different time periods. Using the threshold *θ*, we define the correlation index *γ*,

17 $${\gamma }_{{t}_{m},{t}_{n}}=(\begin{array}{ll}\mathrm{0,} & {d}_{{t}_{m},{t}_{n}}\ge \theta ,\\ 1-{d}_{{t}_{m},{t}_{n}}/\theta , & {d}_{{t}_{m},{t}_{n}} < \theta \mathrm{.}\end{array}$$

Here ${\gamma }_{{t}_{m},{t}_{n}}$ is the correlation degree of LPHVG at time *t*_*m*_ and time *t*_*n*_, and ${\gamma }_{{t}_{m},{t}_{n}}$ can be visualized using a recursive graph constructed using the formula

18 $$\Re ({t}_{m},{t}_{n})={\rm{\Theta }}(\theta -d[LPHVG({t}_{m}),LPHVG({t}_{n})]),\quad {\rm{\Theta }}(x)=(\begin{array}{ll}\mathrm{1,} & x > \mathrm{0,}\\ \mathrm{0,} & x\le 0.\end{array}$$

where Θ(*x*) is the Heaviside function. We use the formula to plot the dependence between *LPHVG*(*t*_*m*_) and *LPHVG*(*t*_*n*_) in two-dimensional coordinates, in which both the abscissa and the ordinate are the time *t*. In the recursive graph when the Euclidean distance between *LPHVG*(*t*_*m*_) and *LPHVG*(*t*_*n*_) is sufficiently close, i.e., when $\Re ({t}_{m},{t}_{n})=1$, we plot a dot at (*t*_*m*_, *t*_*n*_) and (*t*_*n*_, *t*_*m*_). Note that at (*t*_*m*_, *t*_*m*_) and (*t*_*n*_, *t*_*n*_), i.e., the dots remain in the main diagonal (Fig. 8), and we can use this to characterize the global dynamic changes in correlation.

### Data Availability

All data generated or analysed during this study are included in this published article.

## Electronic supplementary material

Supplementary Information
