# Supplementary material for: Exact results of the limited penetrable horizontal visibility graph associated to random time series and its application
Source: Sci Rep. 2018 Mar 23;8:5130. doi: 10.1038/s41598-018-23388-1 (PMC5865175; doi:10.1038/s41598-018-23388-1)
Supplement: Supplementary file 1 — Supplementary Information [file 41598_2018_23388_MOESM1_ESM.pdf]

Supplementary Information

Exact results of the limited penetrable horizontal visibility graph associated to random time series and its application

Minggang Wang,[1,2,3] André L.M.Vilela,[3,4] Ruijin Du,[3,5] Longfeng Zhao,[3] Gaogao Dong,[3,5] Lixin Tian,[1,5,*] and H. Eugene Stanley[3]

[1] *School of Mathematical Science, Nanjing Normal University, Nanjing 210042, Jiangsu, China*

[2] *Department of Mathematics, Nanjing Normal University Taizhou College, Taizhou 225300, Jiangsu, China*

[3] *Center for Polymer Studies and Department of Physics, Boston University, Boston, MA 02215, USA*

[4] *Universidade de Pernambuco, 50720-001, Recife-PE, Brazil*

[5] *Energy Development and Environmental Protection Strategy Research Center, Jiangsu University, Zhenjiang, 212013 Jiangsu, China*

PACS numbers: 05.45. Tp, 89.75. Hc, 05.45.-a

Theorem S1. Let $X(t)$ be a real bi-infinite time series of $i.i.d.$ random variables with probability density $f(x)$, with $x \in [a, b]$, and consider its associated LPHVG with a limited penetrable distance $\rho = 1$. Then

$$P(k) \sim exp[-(k-4)ln(5/4)], \quad k = 4, 5, ..., \forall f(x).$$

Proof: Using a method similar to that presented in Refs. [10,11], we select a generic datum x_0 to be the seed. We calculate the probability that an arbitrary datum with value x_0 has a limited penetrable visibility of exactly k other data. From the definition of LPHVG, when x_0 has penetrable visibility of k data there will be at least two penetrable data and two bounding data, one penetrable and one bounding datum on the right-hand side of x_0 and one on the left-hand side, such that the $k-4$ remaining visible and penetrable visible data are located inside two bounding data. Note that $k = 4$ is the minimum possible degree (see Fig. S1).

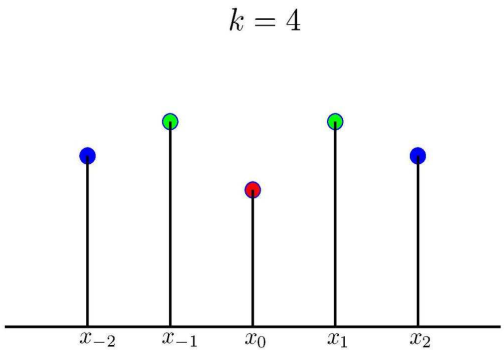

*Electronic address: `tianlx@ujs.edu.cn`

Fig.S1. *Set of possible configuration for a seed data x_0 with $k = 4$. The green dots are penetrable data, the blue dots are bounding data.*

To derive the degree distribution of the associated LPHVG, we first compute some easy terms. Fig. S1 shows the simplest case $P(k = 4)$ in which there are two penetrable data (x_{-1}, x_1) and two bounding data (x_{-2}, x_2). To assure that $k = 4$, we set the height of both the penetrable and bounding data greater than x_0, i.e., $x_{-1} \geq x_0, x_1 \geq x_0$ and $x_{-2} \geq x_0, x_2 \geq x_0$. Then

$$\begin{aligned} &P(k = 4) = Prob(x_{-2}, x_{-1}, x_1, x_2 \geq x_0) \\ &= \int_a^b f(x_0)dx_0 \int_{x_0}^b f(x_{-2})dx_{-2} \int_{x_0}^b f(x_{-1})dx_{-1} \int_{x_0}^b f(x_1)dx_1 \int_{x_0}^b f(x_2)dx_2. \end{aligned} \quad (S1)$$

In order to simplify Eq. (S1), we define the cumulative probability distribution function $F(x)$ of any probability density $f(x)$ to be

$$F(x) = \int_a^x f(t)dt, \quad (S2)$$

where $dF(x)dx = f(x)$,$F(a) = 0$ and $F(b) = 1$. With a loss of generality, we assume $a = 0, b = 1$, i.e., $F(0) = 0$ and $F(1) = 1$. Here the relation between f and F holds, i.e.,

$$\frac{dF^n(x)}{dx} = nf(x)F^{n-1}(x). \quad (S3)$$

Using Eqs. (S2) and (S3), we rewrite Eq. (S1) to be

$$P(k = 4) = \int_0^1 f(x_0)[1 - F(x_0)]^4 dx_0 = \frac{1}{5}, \forall f(x). \quad (S4)$$

When $P(k = 5)$ (see Fig. S2), four configurations emerge: Case 1: C_0^1, in which x_0 has penetrable variables x_{-1} and x_1, bounding variables x_{-2} and x_3, and a right-hand side inner variable x_2. Case 2: C_0^2, in which x_0 has penetrable variables x_{-1} and x_2, bounding variables x_{-2} and x_3, and a right-hand side inner variable x_1. Case 3: C_1^1, in which x_0 has penetrable variables x_{-2} and x_1, bounding variables x_{-3} and x_2, and a left-hand side inner variable x_{-1}. Case 4: C_1^2, in which x_0 has penetrable variables x_{-1} and x_1, bounding variables x_{-3} and x_2, and a left-hand side inner variable x_{-2}.

Thus

$$P(k = 5) = P(C_0^1) + P(C_0^2) + P(C_1^1) + P(C_1^2) \equiv p_0^1 + p_0^2 + p_1^1 + p_1^2. \quad (S5)$$

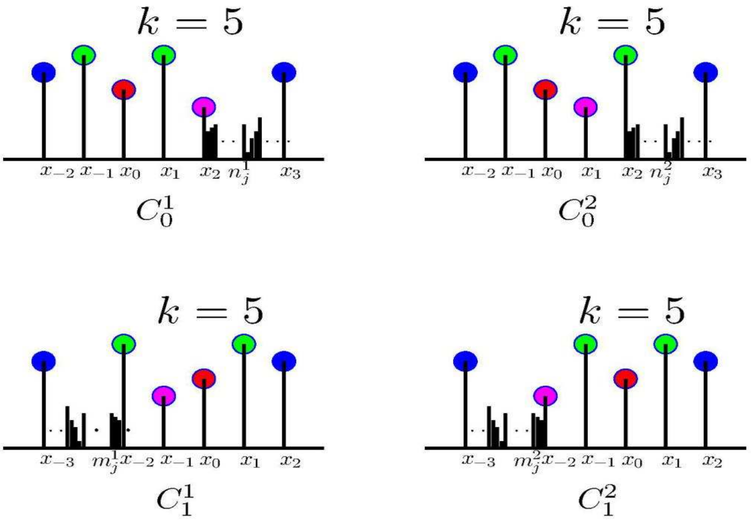

Fig.S2. *Set of possible configurations for a seed data x_0 with $k=5$. The sign of the subscript in x_i indicates whether the data are located on the left-hand side of x_0 or on the right-hand side. The sigh of the subscript in C_i^j indicates the number of inner data located on the left-hand side of x_0, the superscript in C_i^j indicates the different cases. The signs $n_j^1, n_j^2, m_j^1, m_j^2$ indicate the number of the hidden data.*

Note that an arbitrary number of hidden variables $n_j^1, n_j^2, m_j^1, m_j^2$ eventually are located between the inner data and the bounding variables (or the penetrable data and the bounding data) and this must be taken into account in the probability calculation. The geometrical restrictions for the hidden variables are $n_j^1 < x_2, n_j^2 < x_1, j = 1, 2, ..., r$ for C_0^1, C_0^2 and $m_j^1 < x_{-1}, m_j^2 < x_{-2}, j = 1, 2, ..., s$ for C_1^1, C_1^2. Then

$$\begin{aligned} p_0^1 &= Prob((x_{-2}, x_{-1}, x_1, x_3 \geq x_0) \cap (x_2 < x_0) \cap (\{n_j^1 < x_2\}_{j=1,2,...,r})), \\ p_0^2 &= Prob((x_{-2}, x_{-1}, x_2, x_3 \geq x_0) \cap (x_1 < x_0) \cap (\{n_j^2 < x_1\}_{j=1,2,...,r})), \\ p_1^1 &= Prob((x_{-3}, x_{-2}, x_1, x_2 \geq x_0) \cap (x_{-1} < x_0) \cap (\{m_j^1 < x_{-1}\}_{j=1,2,...,s})), \\ p_1^2 &= Prob((x_{-3}, x_{-1}, x_1, x_2 \geq x_0) \cap (x_{-2} < x_0) \cap (\{m_j^2 < x_{-2}\}_{j=1,2,...,s})). \end{aligned} \tag{S6}$$

Because these are independent and identically distributed random variables, p_0^1 can be calculated

$$\begin{aligned} p_0^1 = &\int_0^1 f(x_0)dx_0 \int_{x_0}^1 f(x_{-2})dx_{-2} \int_{x_0}^1 f(x_{-1})dx_{-1} \int_{x_0}^1 f(x_1)dx_1 \int_{x_0}^1 f(x_3)dx_3 \int_0^{x_0} f(x_2)dx_2 \\ &+ \sum_{r=1}^{\infty} \int_0^1 f(x_0)dx_0 \int_{x_0}^1 f(x_{-2})dx_{-2} \int_{x_0}^1 f(x_{-1})dx_{-1} \int_{x_0}^1 f(x_1)dx_1 \int_{x_0}^1 f(x_3)dx_3 \int_0^{x_0} f(x_2)dx_2 \prod_{j=1}^{r} \int_0^{x_2} f(n_j^1)dn_j^1. \end{aligned} \tag{S7}$$

From Eq. (S3) we now have

$$\begin{aligned} p_0^1 &= \int_0^1 f(x_0)dx_0 \int_{x_0}^1 f(x_{-2})dx_{-2} \int_{x_0}^1 f(x_{-1})dx_{-1} \int_{x_0}^1 f(x_1)dx_1 \int_{x_0}^1 f(x_3)dx_3 \int_0^{x_0} \frac{f(x_2)}{1-F(x_2)}dx_2 \\ &= -\int_0^1 f(x_0)dx_0[1-F(x_0)]^4 ln[1-F(x_0)] = \frac{1}{25}. \end{aligned} \tag{S8}$$

Using the same method, we find the identical results for p_0^2, p_1^1 and p_1^2 and then we have

$$P(k=5) = 4P_0^1 = -4\int_0^1 f(x_0)dx_0[1-F(x_0)]^4 ln[1-F(x_0)] = \frac{4}{25}. \tag{S9}$$

We thus conclude that a configuration C_i^j contributes to $P(k)$ with a product of internals when (i) the seed variable $[S]$ provides a contribution of $\int_0^1 f(x_0)dx_0$, (ii) each penetrable variable $[P]$ provides a contribution of $\int_{x_0}^1 f(x)dx$, (iii) each boundary variable $[B]$ provides a contribution of $\int_{x_0}^1 f(x)dx$, and (iv) an inner variable $[I]$ provides a contribution of $\int_{x_j}^{x_0} \frac{f(x)}{1-F(x)}$.

Using these four rules, we formally schematize the probability associated with each configuration. For example, when $k=4$, $P(k)$ has a single contribution p_0 shown in the formal diagram $[B][P][S][P][B]$. When $k=5$, $P(k) = p_0^1 + p_0^2 + p_1^1 + p_1^2$ where p_0^1 is shown in the diagram $[B][P][S][P][I][B]$, p_0^2 is shown in $[B][P][S][I][P][B]$, p_1^1 is shown in $[B][P][I][S][P][B]$, and p_1^2 is shown in $[B][I][P][S][P][B]$. Thus we derive a general expression for $P(k)$ by applying the four rules for the contribution of each $C_i^j, i = 0, 1, 2, ..., j = 1, 2, ...$ When $k=6$, however, there are 13 possible seed data x_0 configurations, and it is labeled C_0^i, C_1^j, C_2^r.

Similar to $P(k=5)$, we derive

$$P(k=6) = \sum_{i=1} p_0^i + \sum_{j=1} p_1^j + \sum_{r=1} p_2^r. \tag{S10}$$

Here C_1^j leads to the same expression as configurations in $k=5$ and thus we can derive p_1^j by applying the four rules. Fig. S3 shows that C_0^i and C_2^r are geometrically different and are formed from a seed x_0 and two penetrable variables. In configurations C_0^4 and C_2^4 there are three penetrable variables, one of which (x_1 in C_0^4 and x_{-1} in C_2^4) is smaller than x_0. When calculating

$P(k)$ the role of this smaller penetrable variable is similar to the inner variable. Thus without loss of generality we refer to this smaller penetrable variable as the inner variable. There are two bounding and two concatenated inner variables, and the concatenated variables produce concatenated integrals.

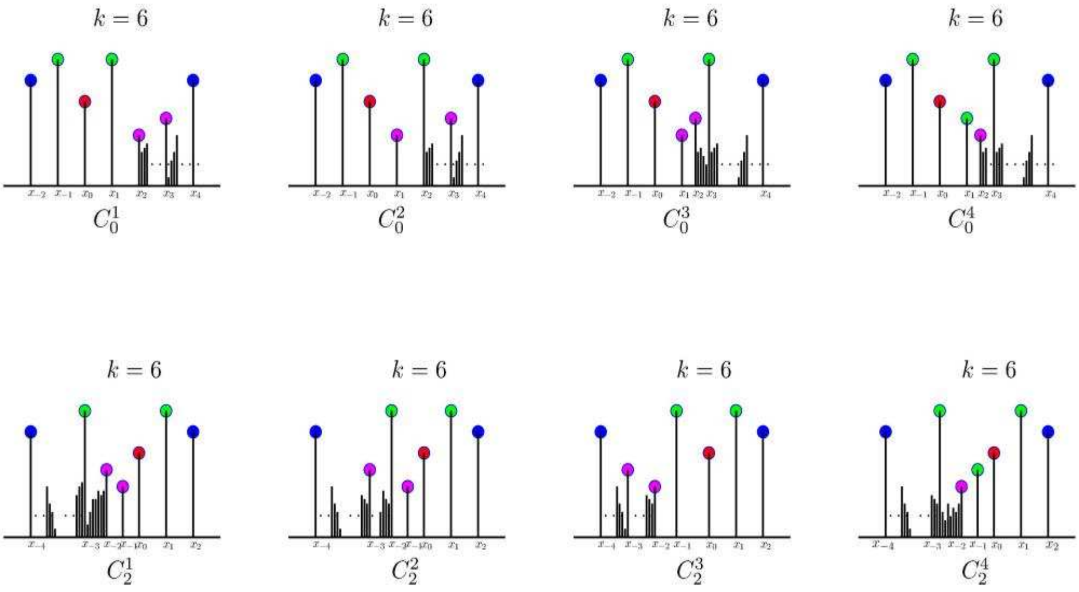

Fig. S3. *Set of possible configurations for* C_0^i, C_2^r *with* $k = 6$.

For example, when we apply the same formalism as for $k = 5$ we find that when $k = 6$, in the case of C_0^i,

$$\begin{aligned}
p_0^1 &= \int_0^1 f(x_0)dx_0 \int_{x_0}^1 f(x_1)dx_1 \int_0^{x_0} \frac{f(x_2)}{1-F(x_2)}dx_2 \int_{x_2}^{x_0} \frac{f(x_3)}{1-F(x_3)}dx_3 \int_{x_0}^1 f(x_4)dx_4 \int_{x_0}^1 f(x_{-1})dx_{-1} \int_{x_0}^1 f(x_{-2})dx_{-2} \\
p_0^2 &= \int_0^1 f(x_0)dx_0 \int_0^{x_0} \frac{f(x_1)}{1-F(x_1)}dx_1 \int_{x_0}^1 f(x_2)dx_2 \int_{x_1}^{x_0} \frac{f(x_3)}{1-F(x_3)}dx_3 \int_{x_0}^1 f(x_4)dx_4 \int_{x_0}^1 f(x_{-1})dx_{-1} \int_{x_0}^1 f(x_{-2})dx_{-2} \\
p_0^3 &= \int_0^1 f(x_0)dx_0 \int_0^{x_0} \frac{f(x_1)}{1-F(x_1)}dx_1 \int_{x_1}^{x_0} \frac{f(x_2)}{1-F(x_2)}dx_2 \int_{x_0}^1 f(x_3)dx_3 \int_{x_0}^1 f(x_4)dx_4 \int_{x_0}^1 f(x_{-1})dx_{-1} \int_{x_0}^1 f(x_{-2})dx_{-2} \\
p_0^4 &= \int_0^1 f(x_0)dx_0 \int_0^{x_0} \frac{f(x_1)}{1-F(x_1)}dx_1 \int_0^{x_1} f(x_2)dx_2 \int_{x_0}^1 f(x_3)dx_3 \int_{x_0}^1 f(x_4)dx_4 \int_{x_0}^1 f(x_{-1})dx_{-1} \int_{x_0}^1 f(x_{-2})dx_{-2}
\end{aligned} \qquad (S11)$$

Using Eq. (S8), when $k = 5$, every integral depends on x_0, and thus we integrate each term to find this dependence on. Here, however, there are two concatenated inner variables, and two concatenated inner variables generate the dependence on the integrals and hence on the probabilities. Thus in the general case each configuration is not equiprobable and does not provide the same contribution to the probability $P(k)$. To weight the effect of these concatenated contributions, we use the definition of p_i. Since $P(k)$ is formed by $k - 3$ contributions labeled $C_0^i, C_1^j, ..., C_{k-4}^r$ in which the subindex denotes the number of inner data present at the left-hand side of seed x_0, we conclude that in general the $k - 4$ inner variables make the following contributions to $P(k)$:

(a) p_0^i has $k - 4$ concatenated internals (the right-hand side of seed x_0);

(b) p_1^j has $k - 5$ concatenated internals (the right-hand side of seed x_0) and an independent inner data contribution (the left-hand side of seed x_0);

(c) p_2^r has $k-6$ concatenated internals (the right-hand side of the seed x_0) and another two independent inner data contributions (the left-hand side of seed x_0);

$\vdots$

(d) p_{k-5}^j has $k-5$ concatenated internals (the left-hand side of seed x_0) and an independent inner data contribution (the right-hand side of seed x_0); and

(e) p_{k-4}^i has $k-4$ concatenated internals (the left-hand side of seed x_0).

Note that p_m^n is symmetric with respect to the seed and the penetrable variables. Adding this modification to the four rules we calculate a general expression for $P(k)$, i.e.,

$$\begin{aligned} P(k) &= \sum_i p_0^i + \sum_j p_1^j + \sum_r p_2^r + ... + \sum_j p_{k-5}^j + \sum_i p_{k-4}^i \\ &= \sum_i [S][P]^2[B]^2[I]_0^i[I]_{k-4}^i + \sum_j [S][P]^2[B]^2[I]_1^j[I]_{k-5}^j + \sum_r [S][P]^2[B]^2[I]_2^r[I]_{k-6}^r + ... \\ &... + \sum_j [S][P]^2[B]^2[I]_{k-5}^j[I]_1^j + \sum_i [S][P]^2[B]^2[I]_{k-4}^i[I]_0^i. \end{aligned} \quad (S12)$$

Using mathematical induction, we prove that

$$P(k) = \sum_{h=0}^{k-4} 3^h [S][P]^2[B]^2[I]_h[I]_{k-4-h}, \quad (S13)$$

where the concatenation of h inner variable integrals $[I]_h$ is

$$[I]_h = \int_0^{x_0} \frac{f(x_1)}{1-F(x_1)} dx_1 \prod_{j=1}^{h-1} \int_{x_j}^{x_0} \frac{f(x_{j+1})}{1-F(x_j+1)} dx_{j+1} = \frac{(-1)^h}{h!}[ln(1-F(x_0))]^h. \quad (S14)$$

Using Eq. (S13) and Eq. (S14), we have

$$\begin{aligned} P(k) &= \sum_{h=0}^{k-4} 3^h \frac{(-1)^{k-4}}{h!(k-4-h)!} \int_0^1 f(x_0)[1-F(x_0)]^4[ln(1-F(x_0))]^{k-4} dx_0 \\ &= (\tfrac{1}{5})^{k-3} \sum_{h=0}^{k-4} \frac{3^h(k-4)!}{h!(k-4-h)!} = \tfrac{1}{5}(\tfrac{4}{5})^{k-4}, \forall f(x). \end{aligned} \quad (S15)$$

Note that $P(k)$ can be rewritten

$$P(k) \sim exp[-(k-4)ln(5/4)], \quad k = 4, 5, 6, ..., \forall f(x). \quad (S16)$$

Theorem S2. Let $X(t)$ be a real valued bi-infinite time series of $i.i.d.$ random variables with a probability density $f(x)$ and with $x \in [a, b]$, and consider its associated LPHVG at a limited penetrable distance ρ. Then

$$P(k) \sim exp\{-(k-2\rho-2)ln[(2\rho+3)/(2\rho+2)]\}, \quad \rho = 0, 1, 2, 3, ... \quad \text{and} \quad k = 2\rho+2, 2\rho+3, ..., \forall f(x).$$

Sketch of the proof. The proof follows a similar path as for LPHVG with the limited penetrable distance $\rho = 1$ (see *Theorem S1*). Instead of Eq. (S13) we now have

$$P(k) = \sum_{h=0}^{k-2(\rho+1)} (2\rho+1)^h [S][P]^{2\rho}[B]^2[I]_h[I]_{k-2(\rho+1)-h}. \quad (S17)$$

We prove by induction that

$$\begin{aligned} P(k) &= \sum_{h=0}^{k-2(\rho+1)} (2\rho+1)^h \frac{(-1)^{k-2(\rho+1)}}{h![k-2(\rho+1)-h]!} \int_0^1 f(x_0)[1-F(x_0)]^{2(\rho+1)}[ln(1-F(x_0))]^{k-2(\rho+1)} dx_0 \\ &= (\tfrac{1}{2\rho+3})^{k-2\rho-1} \sum_{h=0}^{k-2(\rho+1)} \frac{(2\rho+1)^h(k-2(\rho+1))!}{h![k-2(\rho+1)-h]!} = \tfrac{1}{2\rho+3}(\tfrac{2\rho+2}{2\rho+3})^{k-2(\rho+1)}, \forall f(x), \end{aligned} \quad (S18)$$

i.e.,

$$P(k) \sim exp\{-(k-2\rho-2)ln[(2\rho+3)/(2\rho+2)]\}, \quad \rho = 0,1,2,3,... \quad \text{and} \quad k = 2\rho+2, 2\rho+3, ..., \forall f(x). \qquad (S19)$$

When $\rho = 0$ using Eq. (S18) we find $P(k) = \frac{1}{3}(\frac{2}{3})^{k-2}$, the result in Ref. [11]. When $\rho = 0$ the LPHVG becomes the HVG. When $\rho = 1$ the result is the same as in *Theorem S1*.

Theorem S3. Let $X(t)$ be a real valued bi-infinite time series of $i.i.d.$ random variable with probability density $f(x)$ with $x \in [a, b]$, and consider its associated LPHVG with the limited penetrable distance ρ. Then the local clustering coefficient is

$$C_{\min}(k) = \frac{2}{k} + \frac{2\rho(k-2)}{k(k-1)}, \quad \rho = 0,1,2. \quad k \geq 2(\rho+1),$$
$$C_{\max}(k) = \frac{2}{k} + \frac{4\rho(k-3)}{k(k-1)}, \quad \rho = 0,1,2. \quad k \geq 2(2\rho+1).$$

Proof. For a given node x_i, the local clustering coefficient C is the percentage of nodes connected to x_i that are connected to each other. Thus we calculate from a given node x_i the number of nodes from penetrable ρ visible to x_i have mutual penetrable ρ visibility (triangles), normalized with the set of possible triangles $\binom{k}{2}$.

In the simplest $\rho = 1$ case, Fig. S1 shows that when a generic node x_i has a degree $k = 4$ it has two penetrable data and two bounding data, and there are thus five triangles and $C(k = 4) = 5/6$. Fig. S2 shows that when a generic node x_i has a degree $k = 5$ it has two penetrable data, two bounding data, and an inner datum. Here there are two possible outcomes. For configurations C_0^1, C_1^2 there are seven triangles and $C(k = 5) = 7/10$. For configurations C_0^2, C_1^1 there are eight triangles and $C(k = 5) = 8/10$. Fig. S3 shows that when a generic node x_i has a degree $k = 6$ it has two penetrable data, two bounding data, and two inner data. Here there are three possible outcomes. For configuration C_1^3 there are nine triangles and $C(k = 6) = 9/15$. For configurations $C_0^1, C_0^2, C_1^1, C_1^2, C_1^4, C_2^2, C_2^3$ there are 10 triangles and $C(k = 6) = 10/15$. For configurations $C_0^3, C_0^4, C_2^1, C_2^4$ there are 11 triangles and $C(k = 6) = 11/15$. Thus nodes having the same degree can have different clustering coefficients. Although the clustering coefficients of these nodes are irregular, the minimum clustering coefficient and the maximum clustering coefficient are regular.

The calculations of these minimum local clustering coefficients can be rewritten

$$\begin{aligned} C_{\min}(k=4) &= [(k-1)+(k-2)\rho]/\tbinom{k}{2} = 5/6, \\ C_{\min}(k=5) &= [(k-1)+(k-2)\rho]/\tbinom{k}{2} = 7/10, \\ C_{\min}(k=6) &= [(k-1)+(k-2)\rho]/\tbinom{k}{2} = 3/5. \end{aligned} \qquad (S20)$$

In general, for a degree k we can at a minimum form $(k-1)+(k-2)\rho = (1+\rho)k-(2\rho+1)$ triangles out of $\binom{k}{2}$ possibilities, and

$$C_{\min}(k) = [(1+\rho)k-(2\rho+1)]/\tbinom{k}{2} = \frac{2}{k} + \frac{2\rho(k-2)}{k(k-1)}, \quad \rho = 0,1,2. \quad k \geq 2(\rho+1). \qquad (S21)$$

Similarly, the calculation of the maximum local clustering coefficients can be rewritten

$$\begin{aligned} C_{\max}(k=4) &= [(k-1)+2\rho(k-3)]/\tbinom{k}{2} = 5/6, \\ C_{\min}(k=5) &= [(k-1)+2\rho(k-3)]/\tbinom{k}{2} = 8/10, \\ C_{\min}(k=6) &= [(k-1)+2\rho(k-3)]/\tbinom{k}{2} = 11/15. \end{aligned} \qquad (S22)$$

For a degree k we can at a maximum form $(k-1)+2\rho(k-3) = (1+2\rho)k-(6\rho+1)$ triangles out of $\binom{k}{2}$ possibilities, and

$$C_{\max}(k) = [(1+2\rho)k-(6\rho+1)]/\tbinom{k}{2} = \frac{2}{k} + \frac{4\rho(k-3)}{k(k-1)}, \quad \rho = 0,1,2. \quad k \geq 2(2\rho+1). \qquad (S23)$$

This relation between k and $C_{\min}, C_{\max}$ allows us to deduce the local clustering coefficient distribution $P(C_{\min})$ and $P(C_{\max})$,

$$\begin{array}{l} P(k)=\frac{1}{2\rho+3}(\frac{2\rho+2}{2\rho+3})^{k-2(\rho+1)}, \\ k=\frac{\varphi+\sqrt{\varphi^2-8C_{\min}(2\rho+1)}}{2C_{\min}}, \quad \varphi=C_{\min}+2\rho+2, \\ k=\frac{\phi+\sqrt{\phi^2-8C_{\max}(6\rho+1)}}{2C_{\max}}, \quad \phi=C_{\max}+4\rho+2. \end{array} \quad (S24)$$

Then

$$P(C_{\min})=\frac{1}{2\rho+3}exp\{[\frac{\varphi+\sqrt{\varphi^2-8C_{\min}(2\rho+1)}}{2C_{\min}}-2(\rho+1)]ln(\frac{2\rho+2}{2\rho+3})\}, \quad (S25)$$

$$P(C_{\max})=\frac{1}{2\rho+3}exp\{[\frac{\phi+\sqrt{\phi^2-8C_{\max}(6\rho+1)}}{2C_{\max}}-2(\rho+1)]ln(\frac{2\rho+2}{2\rho+3})\}. \quad (S26)$$

Theorem S4. Let $\{x_t\}_{t=0,1,...,n}$ be a bi-finite sequence of $i.i.d.$ random variables extracted from a continuous probability density $f(x)$. Then the probability $P_\rho(n)$ that two data separated by n intermediate data are two connected nodes in the graph is

$$P_\rho(n)=\frac{2\rho(\rho+1)+2}{n(n+1)}, \rho=0,1,2,...$$

Proof. Without loss of generality, we restrict x to $[0,1]$. When $\rho=0$, Ref. [11] derives

$$P_0(n)=\int_0^1\int_0^1 f(x_0)f(x_n)dx_0dx_n\int_0^{min(x_0,x_n)}...\int_0^{min(x_0,x_n)} f(x_1)...f(x_{n-1})dx_1...dx_{n-1}=\frac{2}{n(n+1)}. \quad (S27)$$

When $\rho=1$, because an arbitrary value x_0 from this series will be connected to node x_n if there is no more than one $x_i \geq min(x_0,x_n)$ for all $x_i, i=1,2,...,n-1$. Then $P_1(n)$ is

$$\begin{array}{l} P_1(n)=\int_0^1\int_0^1 f(x_0)f(x_n)dx_0dx_n\int_0^{min(x_0,x_n)}...\int_0^{min(x_0,x_n)} f(x_1)...f(x_{n-1})dx_1...dx_{n-1} \\ +\int_0^1\int_0^1 f(x_0)f(x_n)\int_{min(x_0,x_n)}^1 f(x_1)dx_1\int_0^{min(x_0,x_n)}...\int_0^{min(x_0,x_n)} f(x_2)...f(x_{n-1})dx_2...dx_{n-1} \\ +...+\int_0^1\int_0^1 f(x_0)f(x_n)\int_{min(x_0,x_n)}^1 f(x_{n-1})dx_{n-1}\int_0^{min(x_0,x_n)}...\int_0^{min(x_0,x_n)} f(x_1)...f(x_{n-2})dx_1...dx_{n-2}. \end{array} \quad (S28)$$

Since the integration limits are independent, when we rewrite $x\equiv min(x_0,x_n)$, we have

$$\begin{array}{l} P_1(n)=\int_0^1\int_0^1 f(x_0)f(x_n)F^{n-1}(x)dx_0dx_n+\binom{n-1}{1}\int_0^1\int_0^1 f(x_0)f(x_n)[1-F(x)]F^{n-2}(x)dx_0dx_n \\ =\binom{n-1}{1}\int_0^1\int_0^1 f(x_0)f(x_n)F^{n-2}(x)dx_0dx_n-(n-2)\int_0^1\int_0^1 f(x_0)f(x_n)F^{n-1}(x)dx_0dx_n. \end{array} \quad (S29)$$

Without loss of generality we can fix x_0 and move x_n such that the latter equation becomes

$$\begin{array}{l} P_1(n)=(n-1)[\int_0^1\int_0^{x_0} f(x_0)f(x_n)F^{n-2}(x_n)dx_0dx_n+\int_0^1\int_{x_0}^1 f(x_0)f(x_n)F^{n-2}(x_0)dx_0dx_n] \\ -(n-2)[\int_0^1\int_0^{x_0} f(x_0)f(x_n)F^{n-1}(x_n)dx_0dx_n+\int_0^1\int_{x_0}^1 f(x_0)f(x_n)F^{n-1}(x_0)dx_0dx_n] \\ =\frac{2}{n}-\frac{2(n-2)}{n(n+1)}=\frac{6}{n(n+1)}. \end{array} \quad (S30)$$

When $P_\rho(n), \rho>1$, the calculation follows a path similar to that for $P_1(n)$ such that instead of Eq. (S29) we have

$$P_\rho(n)=\int_0^1\int_0^1 f(x_0)f(x_n)F^{n-1}(x)dx_0dx_n+\binom{n-1}{\rho}\int_0^1\int_0^1 f(x_0)f(x_n)[1-F(x)]^\rho F^{n-(\rho+1)}(x)dx_0dx_n. \quad (S31)$$

Then by induction we prove that

$$P_\rho(n)=\frac{2\rho(\rho+1)+2}{n(n+1)}, \quad \rho=0,1,2,3,... \quad (S32)$$
